# Sensitive Detection of Pharmaceutical Drugs and Metabolites in Serum Using Data-Independent Acquisition Mass Spectrometry and Open-Access Data Acquisition Tools †

**DOI:** 10.3390/ph15070901

**Published:** 2022-07-21

**Authors:** Syed Muhammad Zaki Shah, Arslan Ali, Muhammad Noman Khan, Adeeba Khadim, Mufarreh Asmari, Jalal Uddin, Syed Ghulam Musharraf

**Affiliations:** 1 International Center for Chemical and Biological Sciences, H.E.J. Research Institute of Chemistry, University of Karachi, Karachi 75270, Pakistan; zakihej@gmail.com (S.M.Z.S.); noman5937@gmail.com (M.N.K.); adeeba.abbas@gmail.com (A.K.); 2 Dr. Panjwani Center for Molecular Medicine and Drug Research, International Center for Chemical and Biological Sciences, University of Karachi, Karachi 75270, Pakistan; 3 Department of Pharmaceutical Chemistry, College of Pharmacy, Abha 62529, Saudi Arabia; masmri@kku.edu.sa (M.A.); jalaluddinamin@gmail.com (J.U.); 4 The Affiliated T.C.M Hospital of Southwest Medical University, Luzhou 646099, China

**Keywords:** metabolomics, data-independent acquisition, data-dependent acquisition, MS-DIAL, Skyline, Perseus

## Abstract

Data-independent acquisition (DIA) based strategies have been explored in recent years for improving quantitative analysis of metabolites. However, the data analysis is challenging for DIA methods as the resulting spectra are highly multiplexed. Thus, the DIA mode requires advanced software analysis to facilitate the data deconvolution process. We proposed a pipeline for quantitative profiling of pharmaceutical drugs and serum metabolites in DIA mode after comparing the results obtained from full-scan, Data-dependent acquisition (DDA) and DIA modes. using open-access software. Pharmaceutical drugs (10) were pooled in healthy human serum and analysed by LC-ESI-QTOF-MS. MS1 full-scan and Data-dependent (MS2) results were used for identification using MS-DIAL software while deconvolution of MS1/MS2 spectra in DIA mode was achieved by using Skyline software. The results of acquisition methods for quantitative analysis validated the remarkable analytical performance of the constructed workflow, proving it to be a sensitive and reproducible pipeline for biological complex fluids.

## 1. Introduction

Screening of metabolites and drugs in biological fluids is the utmost challenge due to their low concentration [1]. Screening of biological samples is performed for multiple purposes including metabolomic studies, toxicology, therapeutic compliance testing, doping control, etc. [1]. Because of high sensitivity, specificity, and high-throughput features, liquid chromatography–mass spectrometry (LC-MS) is the most appropriate tool for the qualitative and quantitative analysis of the metabolome [1,2].

Quantification of clinical metabolites based on LC-MS is easily amenable to large-scale studies. This strategy involves analyses of control and case samples followed by the quantification through comparison of corresponding metabolites peak intensities or peak areas [3]. One of the most common methods used for metabolite quantitative profiling involves the analysis of individual biological replicates using the MS1 method for generating quantitative peak areas followed by the analysis of pool samples for MS2-based identification through comparison with tandem mass spectral libraries using data-dependent acquisition (DDA) mode [4]. The quantification at the MS1 level is limited for coeluting isobaric compounds which may require quantification at the MS2 level [4].

However, in DDA methodology, not all precursors became fragmented in a single experiment. Furthermore, the reproducibility of results, as well as sensitively detection of analytes in a complex mixture (as DDA selectively chooses the most abundant metabolite in the mixture), are also the major challenges observed while using the DDA approach [4]. Therefore, to cope with the aforementioned challenges, the data-independent acquisition (DIA) method is developed for quantification. Precursor or data-independent MS2 acquisition methods in LC-MS for the untargeted approach have recently received attention in the field of clinical metabolomics. In contrast to DDA-based MS2 acquisition methods, DIA methods have shown prominence over large coverage of observable molecules, while collecting complete MS2 spectra continuously [5,6]. DIA-based label-free quantification in proteomics has achieved an order of magnitude broader dynamic range, higher reproducibility of identification, and improved sensitivity and accuracy of quantification compared to data-dependent acquisition (DDA)-based proteomics [7]. Therefore, DIA is now considered to be a theoretically more sensitive technique for identification of large as well as small molecules [8,9].

The difficulty with this acquisition mode is the complexity of MS2 data in which all fragment ions are obtained for a large precursor window simultaneously. Moreover, DIA dissociates the link between the molecule’s precursor and its fragments [10]. Therefore, DIA MS2 spectra must be purified from fragment ions of coeluting compounds and noise ions for metabolomic annotations to achieve high overall library-matching scores [10,11].

There is limited open-access software available for efficient use of DDA and DIA datasets for untargeted metabolomic analysis that majorly targets feature generation, normalization, alignment, quantification, transition list generation, and statistical analysis. It is also difficult to perform all these steps on a single open-access software due to a separate algorithm required for each mode. In this context, the aim of this study is to evaluate the performance of MS1 full-scan, DDA, and DIA modes using the easy-to-use pipeline of open-access software for profiling of serum metabolites and quantification of pharmaceutical drugs. For this, a two-step strategy was used. In the first step, MS1 full-scan and data-dependent (MS2) modes were used for feature generation, annotation, and identification using MS-DIAL software from the data acquired on pooled samples. The transition lists were generated containing precursor ions, product ions, and retention time of identified features on MS1 and MS2 levels from both modes. While in the second step, Skyline software was used for deconvolution of MS1/MS2 spectra and quantitative analysis in DIA mode from the data acquired on individual samples.

## 2. Results

We present a simple and effective workflow based on open-access software for simple and comprehensive untargeted analysis including feature generation, identification, quantification, and statistical analysis using MS-DIAL, Skyline, and Perseus. We used 10 drug standards of different concentration levels pooled in the QC sample for three test modes.

**Figure 1:**
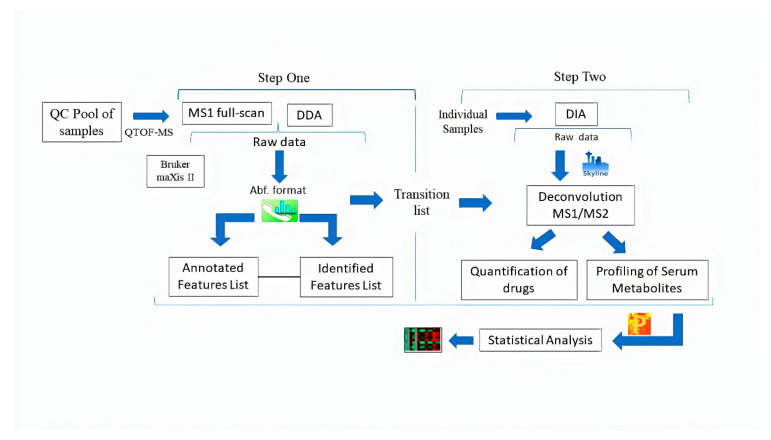
Schematic illustration of the workflow used in this study.

We suggest a two-step strategy. In the first step, feature generation, annotation, identification, and the transition list generation of serum metabolites and drug standards in each diluted point in MS1 full-scan and DDA methods were achieved in MS-DIAL. In the second step, a comprehensive transition list containing precursor ions, product ions, and retention time of each feature imported in Skyline software for processing DIA mode and extracted MS1/MS2 ion chromatograms for deconvolution and quantification. The manual integration where required and usual inspection for features listed on MS1 and MS2 levels were inspected. The peak areas were exported to Perseus software for the statistical analysis. The schematic illustration showed the pipeline of the proposed methodology in Figure 1.

### 2.1. Identification by Using MS-DIAL

In MS-DIAL, features were extracted efficiently by exploring two continuous data axes: retention time (RT) and accurate mass (*m*/*z*) [10]. In total, 372 MS1 features were annotated in the QC sample and exported for the generation of a transition list. A total of 43 features were annotated in DDA mode which were further identified in the LC-MS/MS spectral library (MS/MS-pos), under 5 ppm mass error, and maximum dot products resulted in the identification of 23 serum metabolites and 10 drug standards (Tables S2 and S3 and Figures S1 and S2). The peak areas of each identified feature in each concentration level were used for statistical analysis.

We tried to explore SWATH pipeline available in MS-DIAL for MS2 deconvolution; however, results were not satisfying for DIA MS2 deconvolution. None of the MS2 spectra for the 10 added standards were deconvoluted correctly with their reported fragment ions and retention time (Figure S3). Similarly, serum metabolites identified in DDA were also not correctly deconvoluted in DIA mode using MS-DIAL. This is probably due to the differences in the algorithms used by MS-DIAL for SWATH data compared to Skyline. The transition list of all MS2-acquired features generated from the 100 ng sample in DDA was used for further analysis in DIA mode in Skyline.

### 2.2. Deconvolution of Peak Areas by Using Skyline

**Figure 2:**
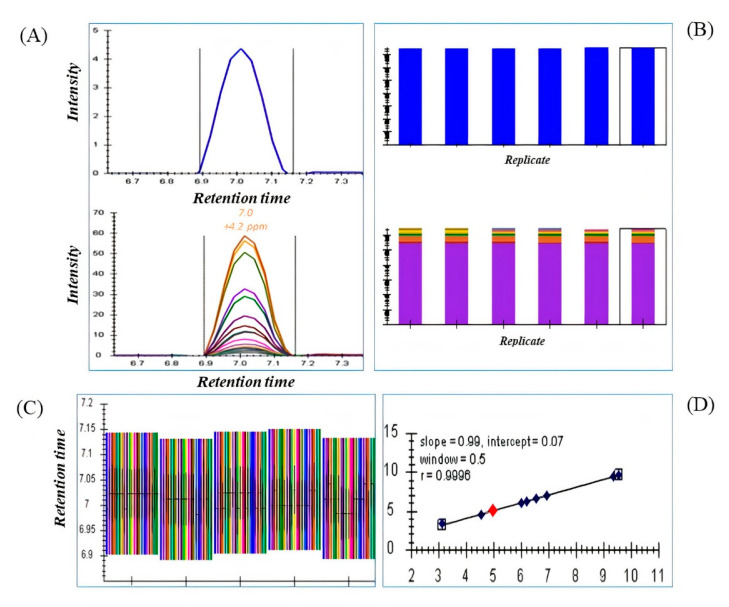
(**A**) Extracted precursor ion chromatogram [top] and fragment ion chromatogram [bottom] of a drug standard dipyridamole (as an example). (**B**). Comparison of peak areas of the precursor [top] and fragment [bottom] ions in multiple samples and replicates. (**C**) The retention time view provides information of each precursor and its fragments present in each serum replicate. (**D**) The calibration curve in the figure proved the consistency and reproducibility of 10 standard drugs in each replicate over the retention time.

A raw transition list from MS1 and DDA containing information of precursor and fragments ions of drugs standard and metabolites generated from MS-DIAL achieved better spectral quality and quantity of MS2 deconvolution in DIA using Skyline. The DIA mode described in this method was used to achieve sensitivity, resolve co-elution and quantification with an isolation window of 25 and 50 Da by automatically extracting MS1 and MS2 ion chromatograms from transition lists. Skyline provides easy observation of retention time, extracted ion chromatograms, and peak areas for each drug and metabolite across numerous samples or replicates as shown in Figure 2. The efficiency of Skyline was tested by using standard drugs at different concentration levels. Quantitative parameters such as LOD, LOQ, and R2 were calculated to compare the quantitative performance of the method (Table 1). All drugs efficiently extracted out and deconvoluted in all samples and were compared for quantification (Figure S1). In total, 23 serum metabolites were also deconvoluted and peak areas were exported for comparison with MS1 and DDA data. All spectral information of DIA data using Skyline is provided in the supplementary information, Figure S4.

### 2.3. Comparison of Quantitative Results

**Figure 3:**
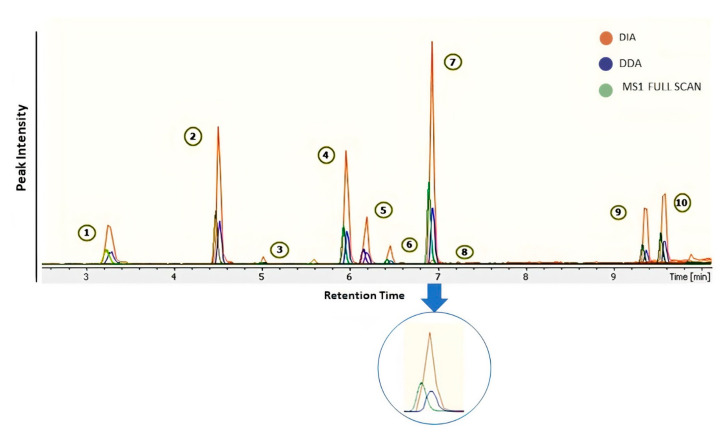
Extracted ion chromatograms of 10 drug standards (**1**. Ranitidine; **2**. Oxytetracycline; **3**. Ranolazine; **4**. Atropine; **5**. Diphenhydramine; **6**. Haloperidol; **7**. Dipyridamole; **8**. Duloxetine; **9**. Finasteride; **10**. Phenylbutazone) in the QC sample and improved sensitivity of drug standards in various modes.

**Figure 4:**
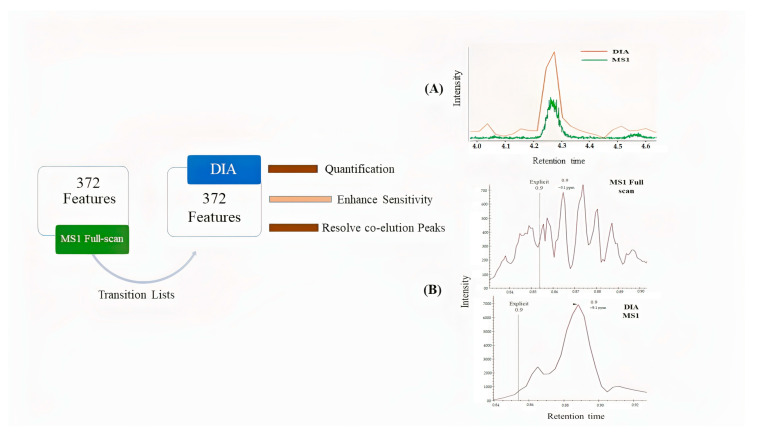
In total, 372 features of the QC sample were annotated using MS1 mode in MS-DIAL, then compared using the DIA mode in Skyline with the transition list (1-naphthylamine, a serum metabolite taken as an example) that resulted in (**A**) enhanced sensitivity in DIA and (**B**) resolved co-elution of peaks and quantitative analysis on the MS1 level in DIA mode.

Skyline was used for extracted ion chromatograms (EICs) of drugs. MS1 full-scan, DDA, and DIA modes were used to compare sensitivity and detection limits on MS1/MS2 levels (Figure 3). The dilution series of QC pool sample ranges from 0.3 to 100 ng mL^−1^ in blood serum were analyzed in three modes. We observed low sensitivity of precursor ion peak areas in DDA mode as compared to two other acquisition modes (Figure 3). Despite the advantage of a small isolation window in DDA (≤1.0 Da) for better MS2 spectral shape, a drawback in sensitivity of peaks was also noticed. MS1 full-scan provides better sensitivity than DDA mode but surprisingly less sensitivity than DIA in all annotated features (Figure 4). Additionally, DIA mode resolved the co-elution problem in many features. Probably, in DIA mode the auto-tune settings allow better transition of ions for MS1 scan; thereby, increasing the peaks significantly.

We compared the sensitivity, detection limit, and reproducibility of three modes (i.e., MS1 full-scan, DDA, and DIA) on MS1 and MS2 levels for DIA data Skyline. Response curves with linear regression were calculated in Microsoft Excel 2016. The linear calibration curve was generated by analyzing five calibration points of blood serum in triplicates. Limit-of-detection (LOD) and limit-of-quantitation (LOQ) for each analyte were calculated through standard error and slope (s).

**Figure 5:**
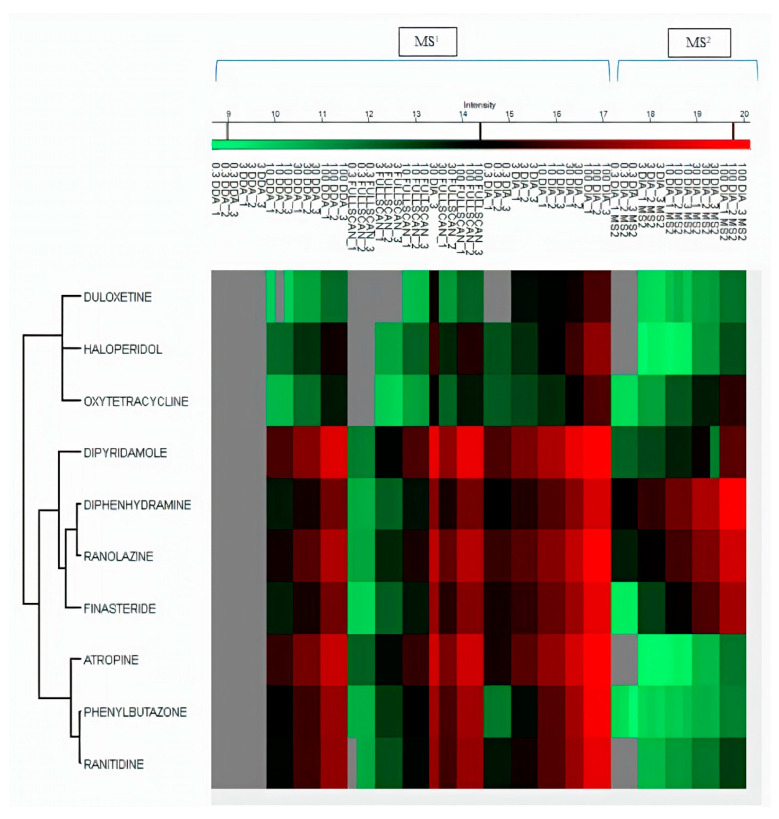
Clustering heatmap of MS1 data of 10 drugs compared between MS1 full scan, DDA, and DIA. DIA MS2 data of all drugs compared between DIA MS1 in all dilution points with replicates.

**Figure 6:**
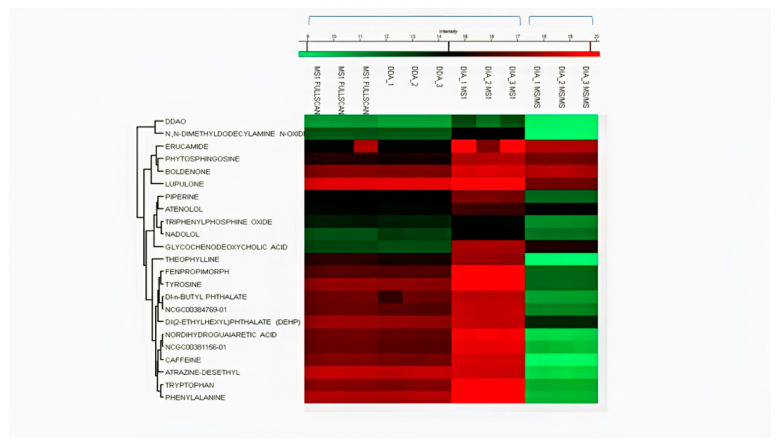
Clustering heatmap of serum metabolites analyzed by MS1 with three replicates in MS1 full-scan, DDA, and DIA modes and DIA MS2 level.

The LOD and LOQ for all the drugs in DIA were found ranging between 0.3–1.05 ng mL^−1^ and 1.01–3.19 ng mL^−1^, respectively. For full scan MS1, LOD and LOQ for all drugs were found ranging between 1.01–6.75 ng mL^−1^ and 3.06–20.48 ng/mL^−1^, respectively. While for the DDA dataset, LOD and LOQ were found in the range of 3.3–8.9 ng mL^−1^ and 10.2–27.0 ng mL^−1^, respectively. We then found LOD and LOQ of MS2 data of the DIA mode to select one fragment of relatively high abundance for quantification. In DIA MS2, 5 out of 10 drugs (ranolazine, diphenhydramine, oxytetracycline, finasteride, and dipyridamole) showed lower LOD and LOQ than their MS1 level. The LOD in DIA mode at MS2 level was found to range between 0.27 and 11.15 ng mL^−1^. All LOD and LOQ values at MS1/MS2 levels of all drugs are summarized in Table 1. MS2 level of quantification is considered more selective and precise as compared to MS1 level [12]. These results indicated better reproducibility and sensitivity in DIA mode by combining MS1/MS2 scans in Skyline. Untargeted and targeted extraction of multiple precursor/fragment ion transitions in Skyline allows better analysis and quantification of desired metabolites [12]. The detection and clustering of all drugs with their MS1 and MS2 datasets represent drug intensities in each sample by three replicates on all calibrated points using Perseus software, as shown in Figure 5. The clustering of serum metabolites with replicates in three acquisition modes on MS1/MS2 levels are presented in the heatmap shown in Figure 6.

## 3. Discussion

In a recent study, Klont et al. [1] have developed a DIA strategy employed for drug analysis in biological fluids on a Q-TOF instrument, providing a new choice for drug metabolomics. In another study Pierre, et al. [13] have developed a workflow on an Orbitrap FusionTM mass spectrometer to compare the efficiency and complementarity of DDA and DIA modes in human plasma samples using MS-DIAL. We proposed a pipeline consisting of three well-known software for mass spectrometry data interpretations, MS-DIAL, Skyline, and Perseus. We examined three acquisition modes using their capabilities to identify a wide range of drugs or metabolites and quantify them in the complex fluid using open-access software.

We suggest the use of MS-DIAL software for feature generation, annotation, identification, and MS2 database search followed by a comprehensive transition list generation using MS1 full-scan and DDA mode in MS-DIAL. This transition list can be uploaded to Skyline for quantification in DIA mode. Perseus software can be used for statistical analysis of drug standards and metabolites in QC sample using the efficiency of MS1 full-scan, DDA, and DIA modes.

In recent comparative studies between full-scan, DDA, and DIA mass spectrometry acquisitions, Guo et al. [14] provided valuable information about maximum features generation, and spectral quality using MS-DIAL software. They suggested that both MS1 and DDA data are efficiently processed by the MS-DIAL; however, in DIA mode the amount of noise was found to be higher than DDA and was not able to efficiently deconvolute the DIA data leading to the serious limitation in the analysis. In our experience of processing data in a DIA workflow using MS-DIAL, this resulted in very high noise in MS2 spectra leading to poor MS2 deconvolution and quality of spectra. MS2 spectra of all features and drug standards were not extracted correctly. To better deconvolute the DIA data, MS-DIAL resolves entangled MS2 spectra in SWATH acquisition (DIA) using a two-step process: precursor-peak spotting followed by MS2-level deconvolution [10]. While Skyline deconvolutes MS2 data from the transition list containing the precursor exact mass, fragment mass, and retention time of every detected feature [15]. Skyline, in proteomics application, can easily import identification results from search engines such as MaxQuant, Proteome Discoverer, etc., for the generation of a transition list. However, in metabolomic studies, this is not well implemented in Skyline. In Skyline, this transition list was used for better MS2 deconvolution quality, quantity, and reproducibility in data-independent acquisition (DIA) mode.

The novelty of the proposed study is to generate a wide range of metabolome transition lists for identification and quantification of metabolites in DIA mode on the MS2 level. The developed transition lists of metabolites have minimized the chance of false positive results in DIA analysis. There are some studies reported regarding the comparison between DDA- and DIA-based mass spectrometry for metabolomics. Some software solutions are also discussed for handling wide range spectral data (MS1/MS2) obtained from DIA as well as DDA workflows such as MS-DIAL, Skyline, Mzmine, and GNPS. No comparative study has been carried out to objectively assess the advantages and limitations of these different tools [14,15,16,17,18]. For feature generation, annotation, and identification of metabolites, many researchers are using MS-DIAL software. In our case, MS-DIAL was used for MS1 full-scan, and DDA mode analysis, but was not found suitable for DIA MS2 deconvolution results. However, Skyline is a very efficient choice for DIA deconvolution for MS2 spectra in many samples as compared to other software results.

Quantification information is important for method validation and reproducibility. In our experiments, the DIA method showed the best quantitative results and the lowest detection limit on MS1 and MS2 levels, followed by full-scan and DDA on the MS1 level. As reported by Guo et al. [14] both MS1 and DIA were better for feature generation than DDA due to loss in intensity at the MS1 level; our results also confirmed this issue. However, it is not clear how at the MS1 level intensities were higher in DIA than MS1 and DDA modes. Quantification at the MS2 level provides an additional advantage for coeluting isobaric and high background metabolites by using specific transition for quantitative analysis. This can be useful for both DDA and DIA modes, but for the comprehensive MS2 data, DDA is generally employed with exclusion criteria. This allows deeper MS2 data for low-abundance metabolites. However, MS2 data cannot be used for quantitative analysis. DIA is thus superior as it can be used for quantification of both MS1 and MS2 levels. The peak areas from Skyline can easily be used for statistical analysis using Perseus software. As Perseus is designed for proteomics data, unlike other commercial software it is optimized for use with large numbers of variables and very powerful software for statistical analysis of high-dimensional data; thus, is also well suited for metabolomics data. Unidentified features can also be uploaded for deconvolution in Skyline if required. However, for validation, each peak must be manually inspected for the correct integration of peak areas. The results of acquisition methods for drug profiling and serum metabolites identification validated the remarkable analytical performance of the constructed workflow, proving it to be a sensitive and reproducible pipeline for biological complex fluids.

## 4. Materials and Methods

### 4.1. Chemicals and Reagents

All chemicals and solvents were purchased commercially. Methanol (Analytical grade) solvent was purchased from Merck K GaA, 64271 (Darmstadt, Germany). Formic acid was purchased from Daejung (Daejung chemicals and Metals Co. Ltd., Siheung-si, Korea). Pharmaceutical drugs standards were obtained from the Drug Bank of Dr. Panjwani Center for Molecular Medicine and Drug Research (PCMD), International Center for Chemical and Biological Sciences (ICCBS), University of Karachi and Searle pharmaceutical Pakistan Ltd. An in-house water purification system (Thermo Scientific, Waltham, MA, USA) was utilized to obtain high-purity water (resistivity 18.1 MΩ cm at 25 °C).

### 4.2. Sample Preparation

The sample preparation strategy was followed as previously reported in the literature with few modifications (Kumari, Sindhia, et al., 2020) [19]. The 5 mL sample of blood was taken from a healthy volunteer after ethical clearance from the local ethical committee by venipuncture and transferred to gel-based Becton–Dickinson (BD) vacutainer tubes (BD Franklin Lakes NJ, USA, REF: 367381), interior coated with silicone for clot activation, then centrifuged (EBA-21, Hettich, Germany) for 5 min at 3500 rpm to separate the serum and prepare a quality control (QC) pool stored at −80 ℃ until further analysis. Then, 1 mg of each drug was dissolved in 1 mL methanol for the preparation of stock solutions separately. A pool of 10 drugs was prepared by mixing equal amounts of standard stock solutions. A sample of 4 µg mL^−1^ dilution was prepared from the pooled stock solution and a series of calibration levels with concentrations of 0.3, 3.0, 10.0, 30.0, and 100.0 ng mL^−1^ were prepared. Furthermore, a 100 µL aliquot of the QC pool of serum samples was spiked in each solution, along with 400 µL of methanol and acetonitrile (5:3) mixture. All the sample solutions were vortexed for 2 min and then for 10 min at room temperature. After which, centrifugation was undertaken for 20 min at 12,000 rpm and the supernatant was collected, dried under vacuum at room temperature, and reconstituted in 200 µL of deionized water.

### 4.3. LC-MS Analysis

All acquisitions were performed using the Bruker maXis II HR-QTOF mass spectrometer (Bremen, Germany), coupled with the Dionex Ultimate 3000 series UPLC system (Thermo Fischer Scientific, Waltham, MA, USA), further facilitated with an auto-sampler, column thermostat, and binary RS pump. For the stationary phase, a Macherey-Nagel™ reverse-phase C_18_ Gravity column (3.0 × 100 mm, 1.8 µm), was used. The mobile phase consisted of 0.1% formic acid in water (A), as well as in methanol (B), starting with 10% B and raised to 90% B in 6.5 min, which was maintained at 90% B for 1.5 min and then decreased back to 10% B in 1.0 min, a 1 min equilibration time was given before and after the gradient. The flow rate was 0.5 mL/min. Furthermore, in positive mode, 5 µL of the pool was injected into LCMS. A capillary voltage of 4500 V, end plate offset of 500 V, drying gas (nitrogen) with flow rate of 10 mL/min, drying gas temperature of 280 °C, and nebulizer gas pressure of 45.0 psi, were optimized for this study. For data-dependent acquisitions (DDA) with auto MS/MS within the mass range of 70–1200 *m*/*z*, the exclusion time of the precursor ion of 5 s and scan speed of 20 Hz were used. All parameters were the same in MS1 full-scan and DDA modes. Data-independent acquisitions (DIA), with a Q1 window of 25 and 50 Da used within the mass range of 99–1200 *m*/*z* (Table S1), were carried out with the collision energy of 30 eV.

### 4.4. Software and Data Processing

The MS1 and DDA data acquired for the QC pool sample in raw data (.d) format was first converted into the Analysis Base File (Abf.) format. MS-DIAL software (version 4.24) was used for feature detection, ion species annotation, compound spectra extraction, and peak alignment between samples and spectral analysis resulting in the transition list containing information from every detected feature such as retention time and precursor *m*/*z*, MS2 ions [10]. Identification of drugs and serum metabolites in the complex mixture was undertaken in MS-DIAL using MS/MS libraries (MS/MS-Pos containing 13,303 unique compounds with 290,915 records) which are openly available.

The MS-DIAL parameters were set as follows: retention time 1–13 min; mass range 70–1200 Da; MS1 and MS2 tolerance were 0.01 and 0.05 Da, respectively; minimum peak width, 5 scans; minimum peak height, 1000 amplitude; smoothing level, 2; MS/MS abundance cut off, 10 amplitude; mass slice width, 0.1 Da; exclusion mass list, none; retention time tolerance, 2 min; MS1 accurate mass tolerance, 0.01 Da; MS2 accurate mass tolerance, 0.05 Da; identification score cut off, 70%. Spectral library, (MS/MS-pos) LC-MS/MS in MSP format. For data-independent acquisition, all parameters were the same except for the experimental file containing the DIA window mass range Q1 window of 25 and 50 Da; (Table S1) minimum peak height, 1000; minimum peak width, 4; MS/MS abundance cut off, 5 amplitude.

Skyline (version 21.2.0.425) is open-source software well known in proteomics for its unique features, especially visualization and advanced method refinement options such as retention time scheduling and reproducibility [20,21]. These features are now also implemented for small molecule workflows such as pharmaceuticals (drug metabolites and toxicology), metabolomics, forensics, and food safety [22]. The Skyline parameters were set as follows: TOF for precursor mass analyzer, resolving power: 30,000; mass range: 70–1200 Da; retention time filtering: included all matching scans for MS1 and DDA modes. All parameters remained the same in the DIA mode except for an isolation window with the gap of 50 and 25 Da. covering a mass range of 99.5–1200 Da. for MS2 data. The method has a margin of 0.5 m/z added to the isolation window, for example, 99.5–150.5, 149.5–200.5, etc.

Perseus (version 1.6.2.1), a comprehensive software for functional analysis of high-dimensional proteomics data including data cleansing, preprocessing, exploratory analysis, and statistical methods [23] was used. A CSV file containing peak areas of each replicate was imported by using a generic matrix uploaded in Perseus and the data was transformed to a logarithmic scale (log2(x)). To visualize the sample correlations, the “Analysis, Hierarchical clustering” option was used to cluster all replicates of samples based on the correlation coefficients between them, revealing higher similarity on MS1/MS2 levels.

## 5. Conclusions

In conclusion, we suggest a pipeline that integrates freely available software, MS-DIAL, Skyline, and Perseus for the metabolomic profiling and drug quantification in DIA-based analysis. This will be helpful for the laboratories with limited bioinformatics support, not requiring vendor-specific software for DIA data analysis. MS-DIAL can be used for comprehensive feature generation and transitions using MS1 and DDA data followed by the DIA data deconvolution using a metabolic library of transitions and precursor masses can be superior to MS1-based analysis. This provides an additional advantage to utilizing both MS1 and MS2 levels of quantification using Skyline. In Skyline, the choice of MS1 and MS2 can be made for the quantification of metabolites by visible inspection, allowing for better quantification performance. The peak area comparison using extracted data from MS-DIAL and Skyline in CSV format can be further processed with Perseus statistical software. The proposed strategy can utilize and combine the advantage of MS1, DDA, and DIA acquisitions using open-access software for metabolites profiling and quantification in biological fluids.

## Figures and Tables

**Table 1:** Pharmaceutical drug quantification results by comparing LOD/LOQ for MS1 full-scan, DDA, and DIA modes on MS1 level and LOD/LOQ data of drugs on MS2 level in DIA.

Drugs	Linear Calibration Range (ng/mL)	Acquisitions	Regression Equation	R^2^	LOD (ng/mL)	LOQ (ng/mL)
Ranitidine	0.3–100 10–100 0.3–100 3–100	MS SCAN DDA DIA DIA (MS2)	y = 1094.9x + 686.35 y = 2438.7x + 31,531 y = 3475.3x + 8773 y = 39.904x + 610.2	0.9998 0.9988 0.9999 0.9993	1.23 6.9 0.50 1.4	3.75 21.01 1.51 4.25
Ranolazine	0.3–100 10–100 0.3–100 0.3–100	MS SCAN DDA DIA DIA (MS2)	y = 1704.3x + 1808.7 y = 5460x + 60,586 y = 5792.1x + 29,669 y = 2409.6x + 8245.7	0.9998 0.9975 0.9999 0.9999	1.39 8.9 1.00 0.27	4.23 27.02 3.03 3.66
Diphenhydramine	0.3–100 10–100 0.3–100 0.3–100	MS SCAN DDA DIA DIA (MS2)	y = 682.45x + 426.99 y = 1857.6x + 25,226 y = 2538x + 14,939 y = 4992.9x + 11,820	0.9998 0.9994 0.9999 0.9999	1.18 4.7 1.0 0.55	3.58 14.3 2.94 11.04
Phenylbutazone	0.3–100 10–100 0.3–100 0.3–100	MS SCAN DDA DIA DIA (MS2)	y = 1297.7x + 508.63 y = 4897.3x − 2071.5 y = 4780x − 2760.8 y = 12.595x + 354.43	0.9998 0.9997 0.9999 0.9988	2.72 3.63 1.0 3.10	8.25 11.08 3.19 9.33
Oxytetracycline	3–100 10–100 0.3–100 0.3–100	MS SCAN DDA DIA DIA (MS2)	y = 73.192x + 260.82 y = 382.84x + 2845.8 y = 383.1x + 2744.1 y = 265.17x + 307.1	0.9998 0.9997 0.9999 0.9999	1.85 3.32 0.65 0.29	5.60 10.21 1.97 0.90
Duloxetine	10–100 10–100 0.3–100 3–100	MS SCAN DDA DIA DIA (MS2)	y = 21.694x + 427.04 y = 285.82x + 9676.9 y = 291.66x + 9101.4 y = 17.12x + 402.22	0.9989 0.9988 0.9999 0.9918	6.75 6.84 1.0 11.15	20.48 20.73 3.19 19.24
Haloperidol	3–100 10–100 0.3–100 3–100	MS SCAN DDA DIA DIA (MS2)	y = 227.85x + 169.38 y = 900.54x + 9838.5 y = 972.34x + 3121.6 y = 34.018x + 151.12	0.9997 0.9996 0.9999 0.9999	2.06 4.02 0.3 0.68	6.2 12.20 1.01 2.07
Finasteride	0.3–100 10–100 0.3–100 0.3–100	MS SCAN DDA DIA DIA (MS2)	y = 865.32x − 89.72 y = 3613.3x + 7669 y = 3469.7x + 19,199 y = 1366.3x + 553.75	0.9997 0.9995 0.9999 0.9999	1.55 3.65 0.92 0.39	4.69 10.11 2.77 1.44
Dipyridamole	0.3–100 10–100 0.3–100 0.3–100	MS SCAN DDA DIA DIA (MS2)	y = 3700.3x + 1016.8 y = 1346.4x − 13,657 y = 11171x + 42,330 y = 449.73x + 2689.4	0.9999 0.9988 0.9999 0.9999	1.07 4.06 0.83 0.30	3.25 12.4 2.51 0.98
Atropine	0.3–100 10–100 0.3–100 3–100	MS SCAN DDA DIA DIA (MS2)	y = 2458.2x + 3144 y = 7855.3x + 32,350 y = 8014.1x + 21,661 y = 17.495x + 111.04	0.9999 0.9996 0.9999 0.9993	1.01 4.02 0.9 3.32	3.06 12.18 2.98 10.08

## Data Availability

Data are contained within the article and Supplementary Materials.

## Supplementary Materials

The following supporting information can be downloaded at: https://www.mdpi.com/article/10.3390/ph15070901/s1, Table S1. DIA method Q1 window of 25 and 50 Da. Table S2: The 10 pharmaceutical drugs identification using their spectral dot product score using DDA mode in MS-DIAL. Table S3: The 23 serum metabolites identification by their spectral dot product score using DDA mode in MS-DIAL. Figure S1. Identification of all drugs (A. Diphenhydramine, B. Atropine, C. Duloxetine, D. Phenylbutazone, E. Ranitidine, F. Finasteride, H. Ranolazine, G. Haloperidol, I. Oxytetracycline, J. Dipyridamole) have done by using MS-DIAL software in DDA mode. All identified and matched MS2 spectral information under 5ppm mass difference has given below. A transition list generated from all drugs spectral information and extracted in DIA mode using Skyline software. Figure S2. Identified serum metabolites by DDA mode in MS-DIAL. Figure S3. DIA MS2 deconvolution results of drug standards in MS-DIAL. Figure S4. Identified serum metabolites deconvoluted MS1 (top) and MS2 (bottom) spectra by DIA mode in Skyline.

## References

[1] Klont F., Jahn S., Grivet C., König S., Bonner R., Hopfgartner G. (2020). SWATH data independent acquisition mass spectrometry for screening of xenobiotics in biological fluids: Opportunities and challenges for data processing. Talanta.

[2] Yin Y., Wang R., Cai Y., Wang Z., Zhu Z.-J. (2019). DecoMetDIA: Deconvolution of multiplexed MS/MS spectra for metabolite identification in SWATH-MS-based untargeted metabolomics. Anal. Chem..

[3] Kenar E., Franken H., Forcisi S., Wörmann K., Häring H.-U., Lehmann R., Schmitt-Kopplin P., Zell A., Kohlbacher O. (2014). Automated label-free quantification of metabolites from liquid chromatography–mass spectrometry data. Mol. Cell. Proteom..

[4] Zha H., Cai Y., Yin Y., Wang Z., Li K., Zhu Z.-J. (2018). SWATHtoMRM: Development of high-coverage targeted metabolomics method using SWATH technology for biomarker discovery. Anal. Chem..

[5] Li H., Cai Y., Guo Y., Chen F., Zhu Z.-J. (2016). MetDIA: Targeted metabolite extraction of multiplexed MS/MS spectra generated by data-independent acquisition. Anal. Chem..

[6] Pappireddi N., Martin L., Wühr M. (2019). A review on quantitative multiplexed proteomics. ChemBioChem.

[7] Bilbao A., Varesio E., Luban J., Strambio-De-Castillia C., Hopfgartner G., Müller M., Lisacek F. (2015). Processing strategies and software solutions for data-independent acquisition in mass spectrometry. Proteomics.

[8] Malipatil N., Fachim H.A., Siddals K., Geary B., Wark G., Porter N., Anderson S., Donn R., Harvie M., Whetton A.D. (2019). Data independent acquisition mass spectrometry can identify circulating proteins that predict future weight loss with a diet and exercise programme. J. Clin. Med..

[9] Zhou J., Li Y., Chen X., Zhong L., Yin Y. (2017). Development of data-independent acquisition workflows for metabolomic analysis on a quadrupole-orbitrap platform. Talanta.

[10] Tsugawa H., Cajka T., Kind T., Ma Y., Higgins B., Ikeda K., Kanazawa M., VanderGheynst J., Fiehn O., Arita M. (2015). MS-DIAL: Data-independent MS/MS deconvolution for comprehensive metabolome analysis. Nat. Methods.

[11] Röst H.L., Rosenberger G., Navarro P., Gillet L., Miladinović S.M., Schubert O.T., Wolski W., Collins B.C., Malmström J., Malmström L. (2014). OpenSWATH enables automated, targeted analysis of data-independent acquisition MS data. Nat. Biotech..

[12] Li Z., Li Y., Chen W., Cao Q., Guo Y., Wan N., Jiang X., Tang Y.J., Wang Q., Shui W. (2017). Integrating MS1 and MS2 scans in high-resolution parallel reaction monitoring assays for targeted metabolite quantification and dynamic 13C-labeling metabolism analysis. Anal. Chem..

[13] Barbier Saint Hilaire P., Rousseau K., Seyer A., Dechaumet S., Damont A., Junot C., Fenaille F. (2020). Comparative evaluation of data dependent and data independent acquisition workflows implemented on an orbitrap fusion for untargeted metabolomics. Metabolites.

[14] Guo J., Huan T. (2020). Comparison of Full-Scan, Data-Dependent, and Data-Independent Acquisition Modes in Liquid Chromatography–Mass Spectrometry Based Untargeted Metabolomics. Anal. Chem..

[15] Van der Laan T., Boom I., Maliepaard J., Dubbelman A.C., Harms A.C., Hankemeier T. (2020). Data-independent acquisition for the quantification and identification of metabolites in plasma. Metabolites.

[16] Pezzatti J., González-Ruiz V., Boccard J., Guillarme D., Rudaz S. (2020). Evaluation of different tandem MS acquisition modes to support metabolite annotation in human plasma using ultra high-performance liquid chromatography high-resolution mass spectrometry for untargeted metabolomics. Metabolites.

[17] Ten-Doménech I., Martínez-Sena T., Moreno-Torres M., Sanjuan-Herráez J.D., Castell J.V., Parra-Llorca A., Vento M., Quintás G., Kuligowski J. (2020). Comparing targeted vs. untargeted ms2 data-dependent acquisition for peak annotation in LC–MS metabolomics. Metabolites.

[18] Kumari S., Ali A., Roome T., Razzak A., Iqbal A., Siddiqui A.J., Azam S.M.Z., Shaikh H., El-Seedi H.R., Musharraf S.G. (2020). Metabolomics Approach to Understand the Hepatitis C Virus Induced Hepatocellular Carcinoma using LC-ESI-MS/MS. Arab. J. Chem..

[19] Pino L.K., Searle B.C., Bollinger J.G., Nunn B., MacLean B., MacCoss M.J. (2020). The Skyline ecosystem: Informatics for quantitative mass spectrometry proteomics. Mass Spectrom. Rev..

[20] Treutler H., Tsugawa H., Porzel A., Gorzolka K., Tissier A., Neumann S., Balcke G.U. (2016). Discovering regulated metabolite families in untargeted metabolomics studies. Anal. Chem..

[21] Adams K.J., Pratt B., Bose N., Dubois L.G., John-Williams L.S., Perrott K.M., Ky K., Kapahi P., Sharma V., MacCoss M.J. (2020). Skyline for Small Molecules: A Unifying Software Package for Quantitative Metabolomics. J. Proteome Res..

[22] Rardin M.J. (2018). Rapid assessment of contaminants and interferences in mass spectrometry data using skyline. J. Am. Soc. Mass Spectrom..

[23] Tyanova S., Cox J. (2018). Perseus: A bioinformatics platform for integrative analysis of proteomics data in cancer research. Cancer Systems Biology.

