# Supplementary material for: Sensitive Detection of Pharmaceutical Drugs and Metabolites in Serum Using Data-Independent Acquisition Mass Spectrometry and Open-Access Data Acquisition Tools
Source: Pharmaceuticals (Basel). 2022 Jul 21;15(7):901. doi: 10.3390/ph15070901 (PMC9317224; doi:10.3390/ph15070901)
Supplement: Supplementary file 1 [file pharmaceuticals-15-00901-s001.zip › pharmaceuticals-1785168-supplementary.pdf]

Article

Sensitive Detection of Pharmaceutical Drugs and Metabolites in Serum Using Data-Independent Acquisition Mass Spectrometry and Open-Access Data Acquisition Tools †

Syed Muhammad Zaki Shah [1], Arslan Ali [2,*], Muhammad Noman Khan [1], Adeeba Khadim [1], Mufarreh Asmari [3], Jalal Uddin [3] and Syed Ghulam Musharraf [1,2,4,*]

[1] International Center for Chemical and Biological Sciences, H.E.J. Research Institute of Chemistry University of Karachi, Karachi 75270, Pakistan; zakihej@gmail.com (S.M.Z.S.); musharraf1977@gmail.com (S.G.M.) noman5937@gmail.com (M.N.K.); adeeba.abbas@gmail.com (A.K.)

[2] Dr. Panjwani Center for Molecular Medicine and Drug Research, International Center for Chemical and Biological Sciences, University of Karachi, Karachi 75270, Pakistan; arslanali1986@gmail.com (A.A)

[3] Department of Pharmaceutical Chemistry, College of Pharmacy, Abha 62529, Saudi Arabia; masmri@kku.edu.sa (M.A.); jalaluddinamin@gmail.com (J.U.)

[4] The Affiliated T.C.M Hospital of Southwest Medical University, Luzhou 646099, China

* Correspondence: arslanali1986@gmail.com or arslan.ali@iccs.edu (A.A.); musharraf1977@yahoo.com or musharraf@iccs.edu (S.G.M.); Tel.: +92-34819010-174 (A.A.); +92-34819010-134 (S.G.M.)

† Authors would like to dedicate this research work to Professor Atta-Ur-Rahman FRS, on his 80th birthday.

SUPPLEMENTARY DATA

Table S1. DIA method Q1 window of 25 and 50 Da
Table S2: 10 pharmaceutical drugs identification by their spectral dot product score using DDA mode in MS-DIAL
Table S3: 23 serum metabolites identification by their spectral dot product score using DDA mode in MS-DIAL
Figure S1 (**A-J**). Identification of all drugs (**A**) Diphenhydramine; (**B**) Atropine; (**C**) Duloxetine; (**D**) Phenylbutazone; (**E**) Ranitidine; (**F**) Finasteride; (**G**) Haloperidol; (**H**) Ranolazine; (**I**) Oxytetracycline; (**J**) Dipyridamole have done by using MS-DIAL software in DDA mode. All identified and matched MS2 spectral information under 5ppm mass difference has given below. A transition list generated from all drugs spectral information and extracted in DIA mode using Skyline software.
Figure S2 (**A-W**). Identified serum metabolites by their MS2 spectra in DDA mode using MS-DIAL software. The software-generated subfigures contain exact mass, retention time and fragments mass of serum metabolites. (**A**) Phenylalanine; (**B**) Di(2-ethylhexyl) phthalate (DEHP); (**C**) Theophylline; (**D**) Tyrosine; (**E**) Atrazine-desethyl; (**F**) Caffeine; (**G**) Tryptophan; (**H**) N, N- Dimethyl dodecyl amine N-oxide; (**I**) Triphenylphosphine oxide; (**J**) Fenpropimorph; (**K**) Phytosphingosine; (**L**) Nadolol; (**M**) DDAO; (**N**) Lupulone; (**O**) Atenolol; (**P**) Di-n-butyl phthalate; (**Q**) Piperine; (**R**) Nordihydroguaiaretic Acid; (**S**) Erucamide; (**T**) NCGC00384769-01; (**U**) NCGC00381156-01; (**V**) Glycochenodeoxycholic Acid; (**W**) Boldenone-Undecylenate.
Figure S3 (**A-E**). DIA MS2 deconvolution results of drug standards were not satisfying for in MS-DIAL. (A) Diphenhydramine; (B) Phenylbutazone; (C) Ranitidine; (D) Ranolazine; (E) Finasteride.
Figure S4 (**A-W**). Identified serum metabolites deconvoluted MS1 (top) and MS2 (bottom) spectra by DIA mode in Skyline. (**A**) Phenylalanine; (**B**) Atrazine-Desethyl; (**C**) Tryptophan; (**D**) Theophylline; (**E**) Caffeine; (**F**) Boldenone-Undecylenate; (**G**) Nadolol; (**H**) DDAO; (**I**) Phytosphingosine; (**J**) N, N-Dimethyl-dodecyl-amine-N-Oxide; (**K**) Piperine; (**L**) NCGC00384769-01; (**M**) NCGC00381156-01; (**N**) Glycochenodeoxycholic Acid; (**O**) Di-N-Butyl Phthalate; (**P**) Nordihydroguaiaretic Acid; (**Q**) Erucamide; (**R**) Di(2-Ethylhexyl) Phthalate; (**S**) Lupulone; (**T**) Atenolol; (**U**) Triphenylphosphine; (**V**) Tyrosine; (**W**) Fenpropimorph.

Table S1. DIA method Q1 window of 25 and 50 Da.

**Experiment**	**MS Type**	**Min *m/z***	**Max *m/z***
0	SCAN	100	1200
1	SWATH	99.5	150.5
2	SWATH	149.5	200.5
3	SWATH	199.5	225.5
4	SWATH	224.5	250.5
5	SWATH	249.5	275.5
6	SWATH	274.5	300.5
7	SWATH	299.5	325.5
8	SWATH	324.5	350.5
9	SWATH	349.5	375.5
10	SWATH	374.5	400.5
11	SWATH	399.5	425.5
12	SWATH	424.5	450.5
13	SWATH	449.5	475.5
14	SWATH	474.5	500.5
15	SWATH	499.5	525.5
16	SWATH	524.5	550.5
17	SWATH	549.5	575.5
18	SWATH	574.5	600.5
19	SWATH	599.5	625.5
20	SWATH	624.5	650.5
21	SWATH	649.5	675.5
22	SWATH	674.5	700.5
23	SWATH	699.5	725.5
24	SWATH	724.5	750.5
25	SWATH	749.5	775.5
26	SWATH	774.5	800.5
27	SWATH	799.5	825.5
28	SWATH	824.5	850.5
29	SWATH	849.5	875.5
30	SWATH	874.5	900.5
31	SWATH	899.5	950.5
32	SWATH	949.5	1,000.50
33	SWATH	999.5	1,050.50
34	SWATH	1,049.50	1,099.50
35	SWATH	1,049.50	1,150.50
36	SWATH	1,199.50	1,200.50

Table S2: 10 pharmaceutical drugs identification based on their spectral dot product score using DDA mode in MS-DIAL.

Drugs	Formula	RT	*m/z* [H]$^+$	Fragments	Dot Product *
Ranitidine	$C_{13}H_{22}N_4O_3S$	3.3	315.1485	270.0921, 285.2793	0.90
Oxytetracycline	$C_{22}H_{24}N_2O_9$	4.0	461.1555	426.118, 283.0600	0.36
Ranolazine	$C_{24}H_{33}N_3O_4$	5.3	428.2544	279.1702, 304.2017	0.97
Atropine	$C_{17}H_{23}NO_3$	4.5	290.1745	261.1675, 274.1371	0.88
Diphenhydramine	$C_{17}H_{21}NO$	5.4	256.1696	238.714, 167.085	0.81
Haloperidol	$C_{21}H_{23}ClFNO_2$	5.7	376.1474	358.1369, 360.1338	0.72
Dipyridamole	$C_{24}H_{40}N_8O_4$	6.1	505.3245	429.2723, 460.2896	0.99
Duloxetine	$C_{18}H_{19}NOS$	6.1	298.1260	279.1624, 265.0204	0.3
Finasteride	$C_{23}H_{36}N_2O_2$	8.1	373.2850	317.2222, 305.2585	0.91
Phenylbutazone	$C_{19}H_{20}N_2O_2$	8.2	309.1598	263.1523, 281.1650	0.93

* Dot product calculates the similarity between the experimental MS^2 spectrum and the reference MS^2 spectrum. [18] MS-DIAL software calculates dot product ranges between 0 to 1 of every feature. If the experimental MS^2 information does not match with a library fragment ion, the dot product of that ion will be zero.

Table S3: 23 serum metabolites identification based on their spectral dot product score using DDA mode in MS-DIAL.

Metabolites	Precursor Ions $[M+H]^+$ *m/z*	Product Ions *m/z*	Dot Product
Phenylalanine	166.0862	149.0022, 150.0631	0.93
Atrazine-desethyl	188.0707	144.0803, 146.0598	0.55
Tryptophan	205.0972	146.0595, 159.0915	0.68
Theophylline	181.0720	161.0207, 149.0023	0.81
Caffeine	195.0878	163.9774, 161.0193	0.93
N, *N*- Dimethyl dodecyl amine *N*-oxide	230.2484	228.1788, 212.2365	0.74
Di-n-butyl phthalate	279.1595	150.0269, 223.0971	0.64
Boldenone-Undecylenate	453.3440	435.3339	0.84
Nadolol	310.2019	251.1280, 281.0028	0.64
DDAO	202.2166	173.9622, 184.2054	0.75
Phytosphingosine	318.3007	256.2639, 318,3537	0.77
Piperine	286.1444	262.0576, 250.9922	072
NCGC00384769-01	415.2124	413.2654, 295.1181	0.82
NCGC00381156-01	437.1943	233.0757	0.81
Glycochenodeoxycholic Acid	450.3224	414.3005, 339.2688	0.61
Nordihydroguaiaretic Acid	301.1414	245.0798, 301.3036	0.88
Erucamide	338.3424	295.1956, 321.3159	0.80
Di(2-ethylhexyl) phthalate (DEHP)	391.2853	167.0343, 150.0265	0.80
Lupulone	413.2673	301.1421, 413.3803	0.85
Atenolol	267.1637	237.0144, 265.1491	0.79
Fenpropimorph	304.2615	214.9186, 302.1462	0.74
Tyrosine	182.0811	165.0547, 147.0442	0.68
Triphenylphosphine oxide	279.0937	201.0484, 149.0238	0.77

(A) DDA DIA

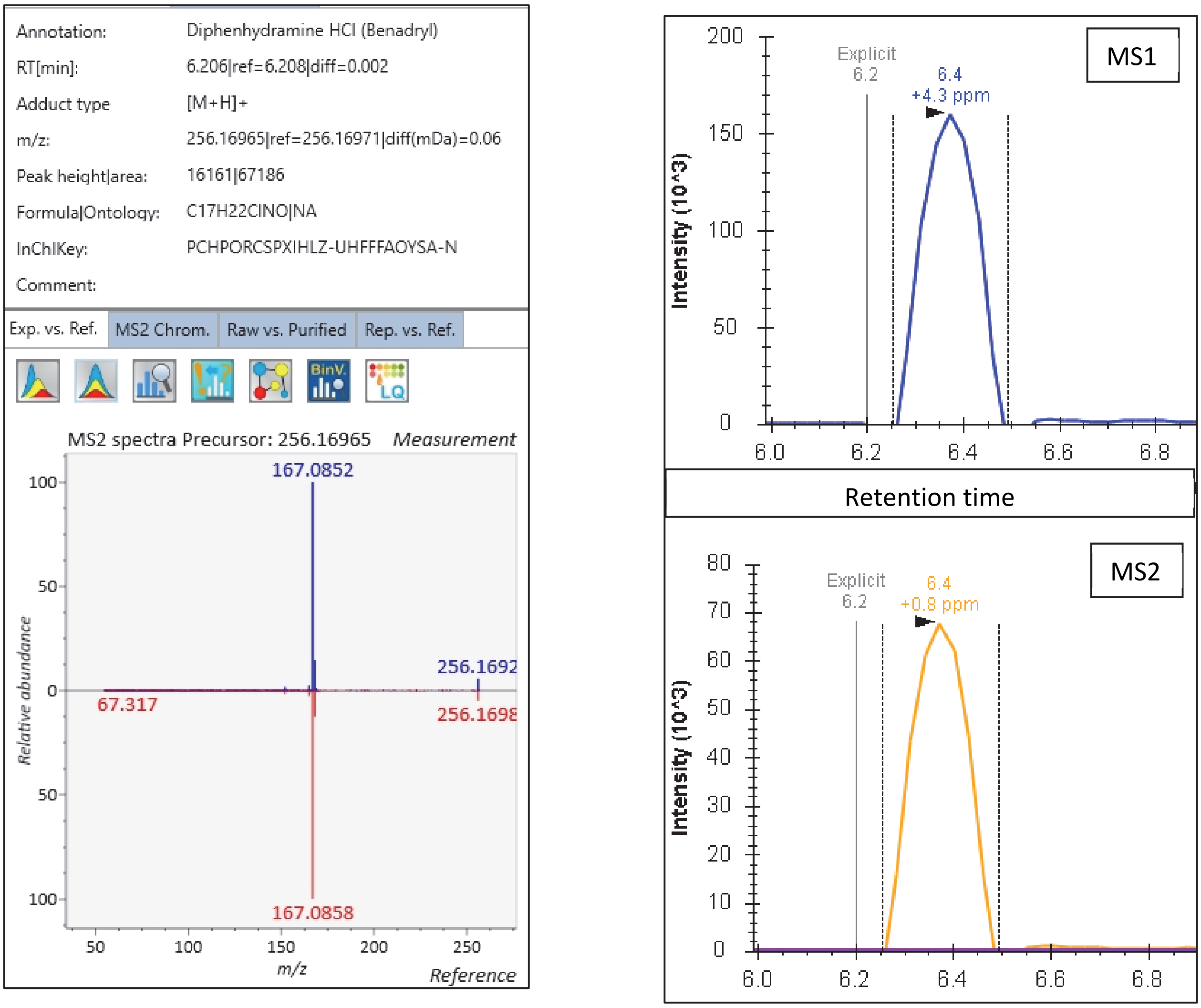

Figure S1. Continue

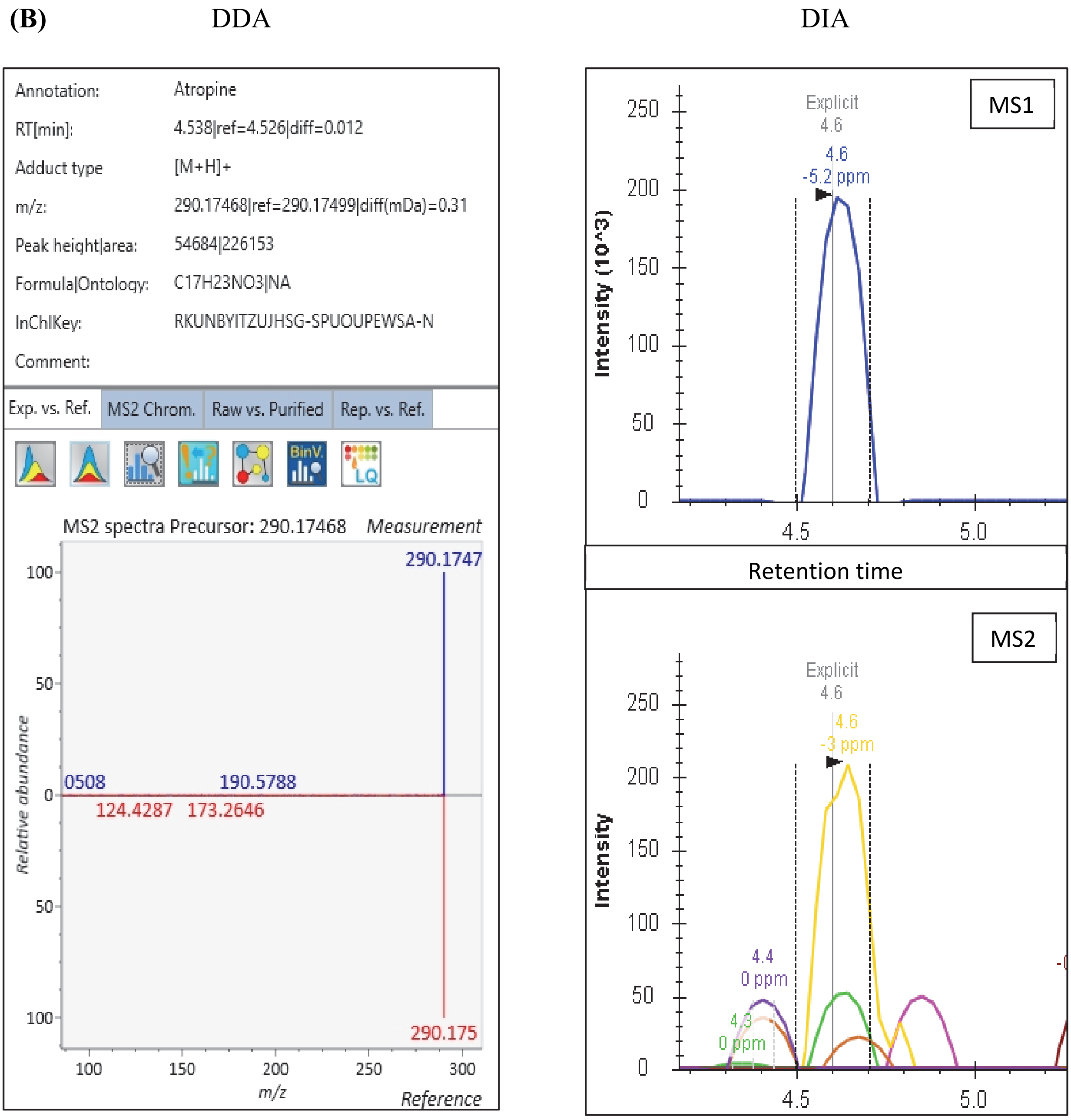

Figure S1. Continue

(**C**) DDA DIA

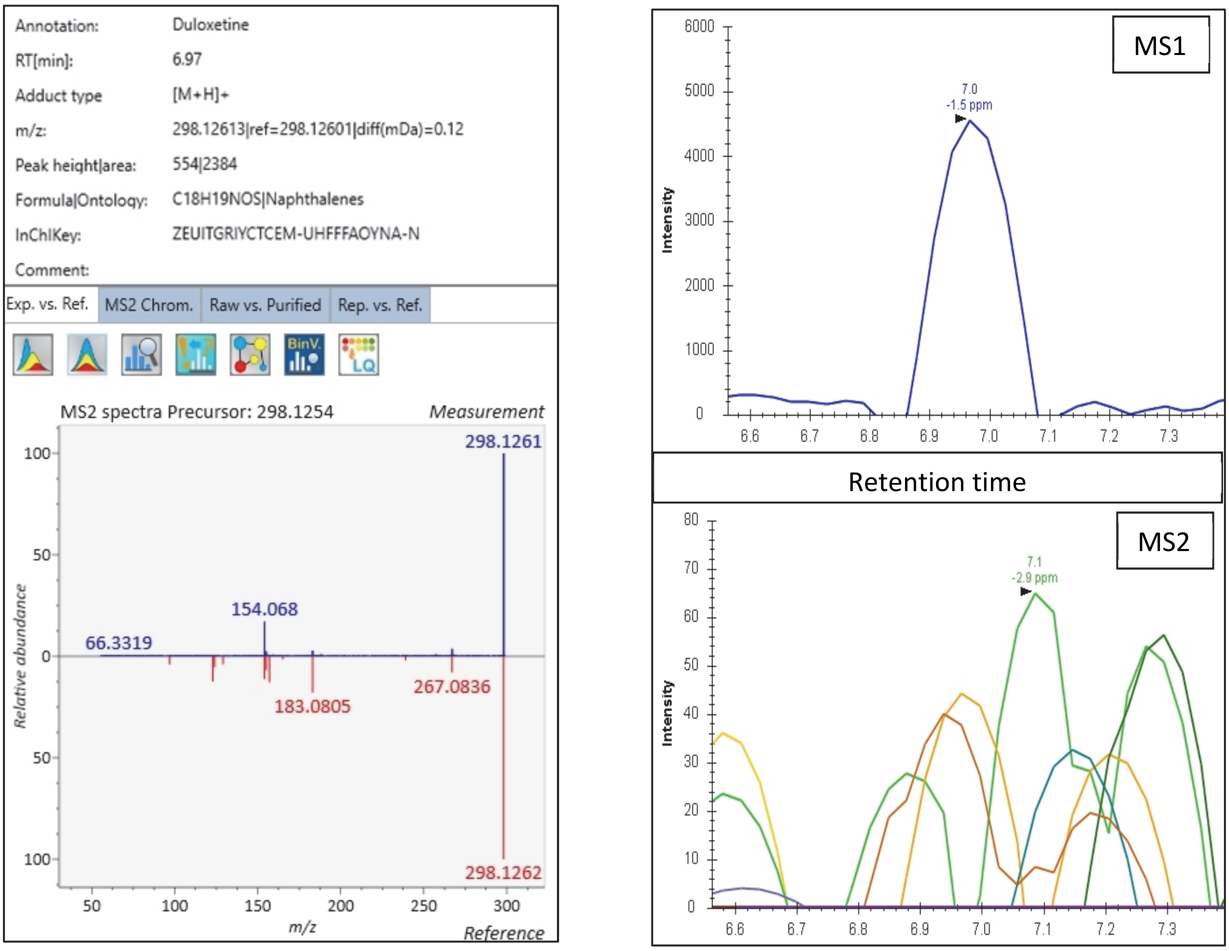

Figure S1. Continue

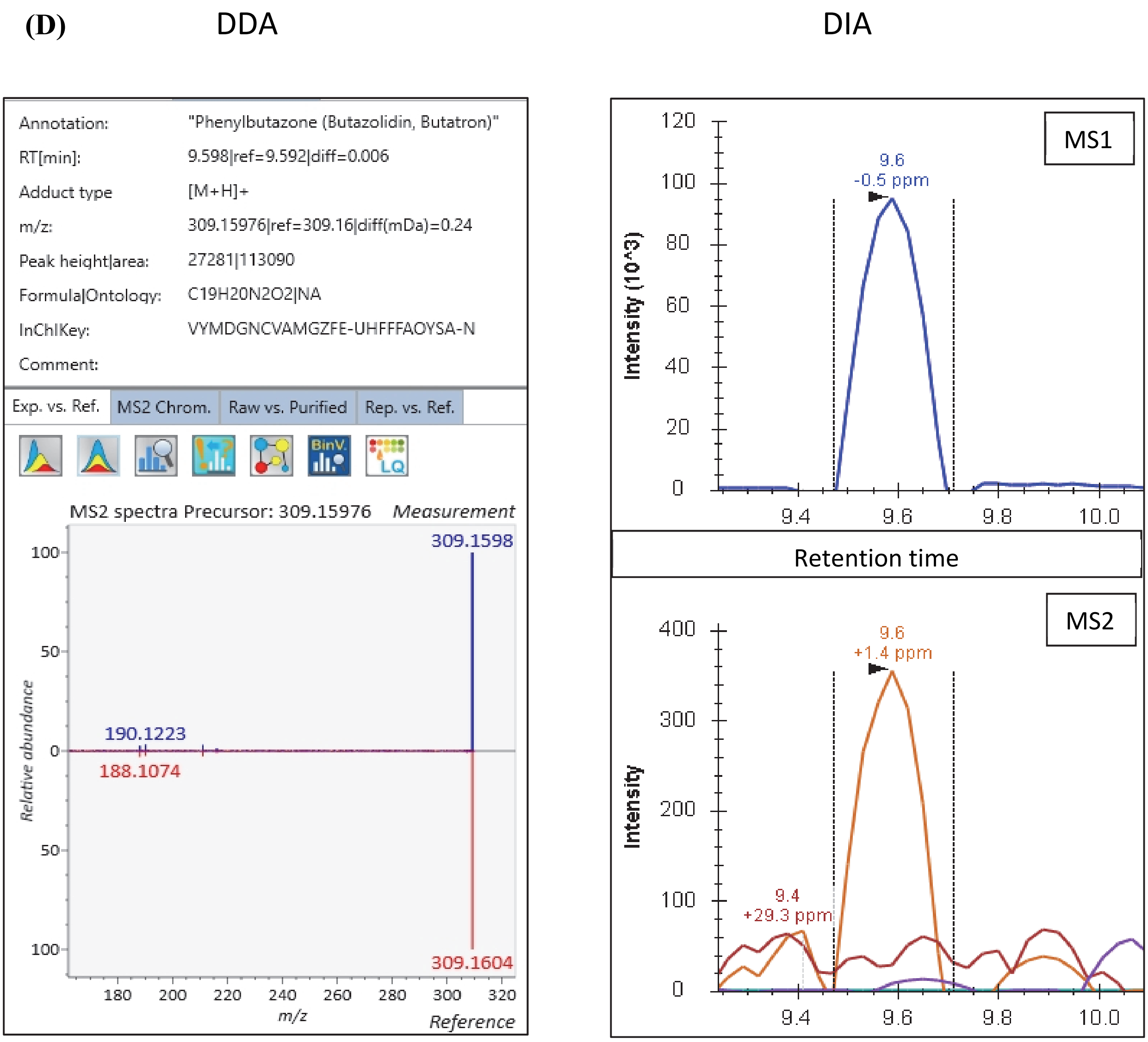

Figure S1. Continue

(E) DDA DIA

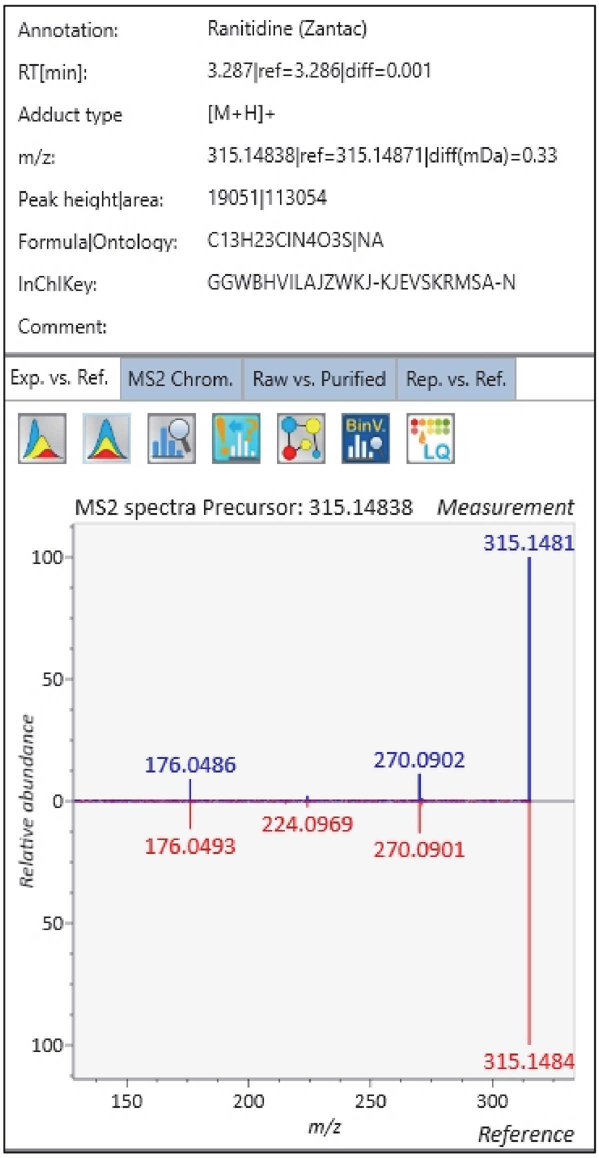

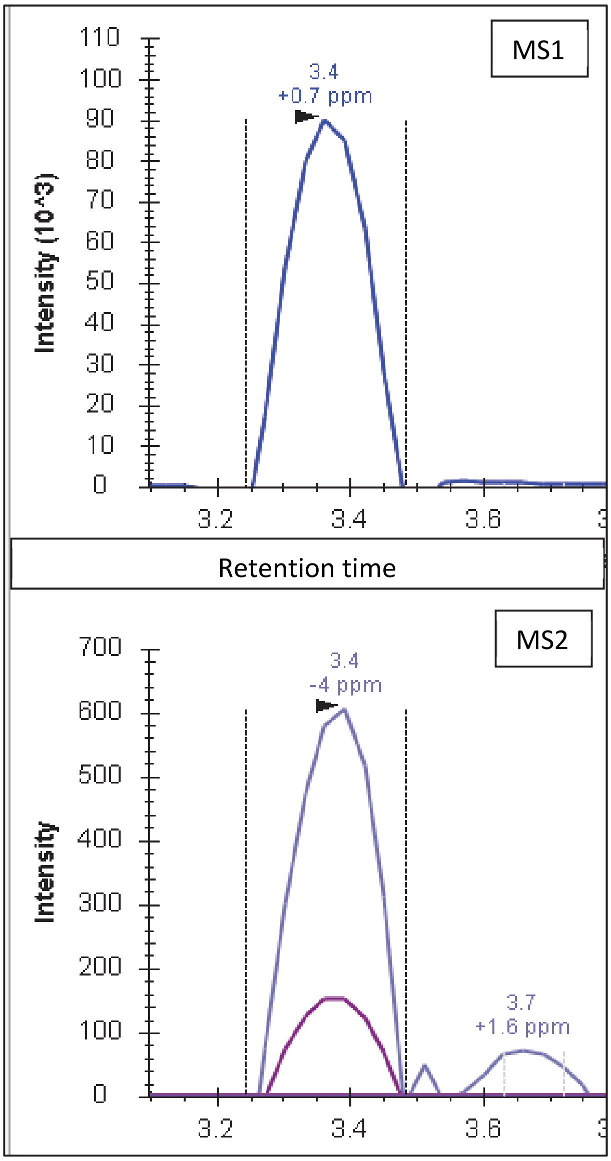

Figure S1. Continue

(F)

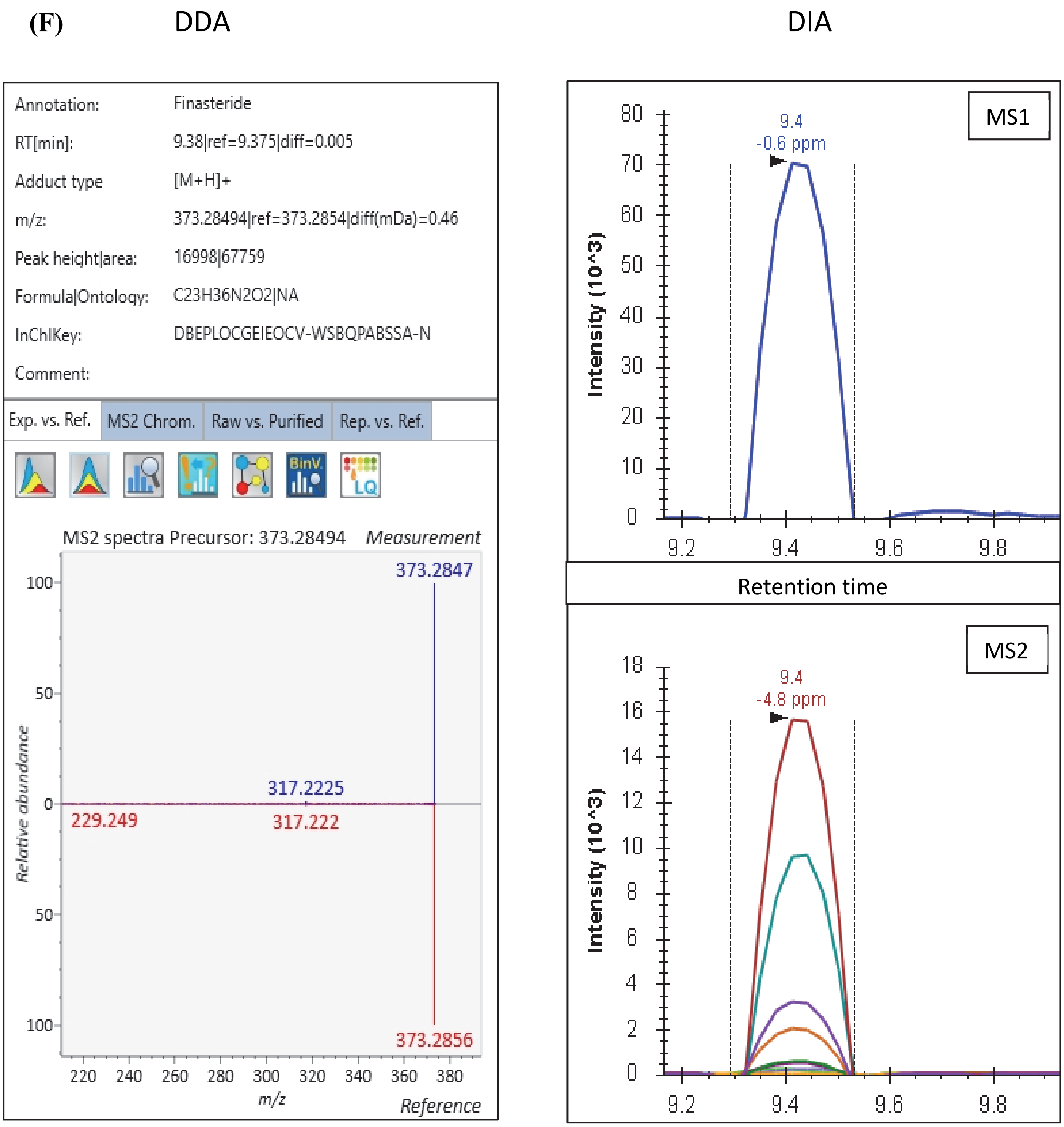

Figure S1. Comtinue

(G) DDA DIA

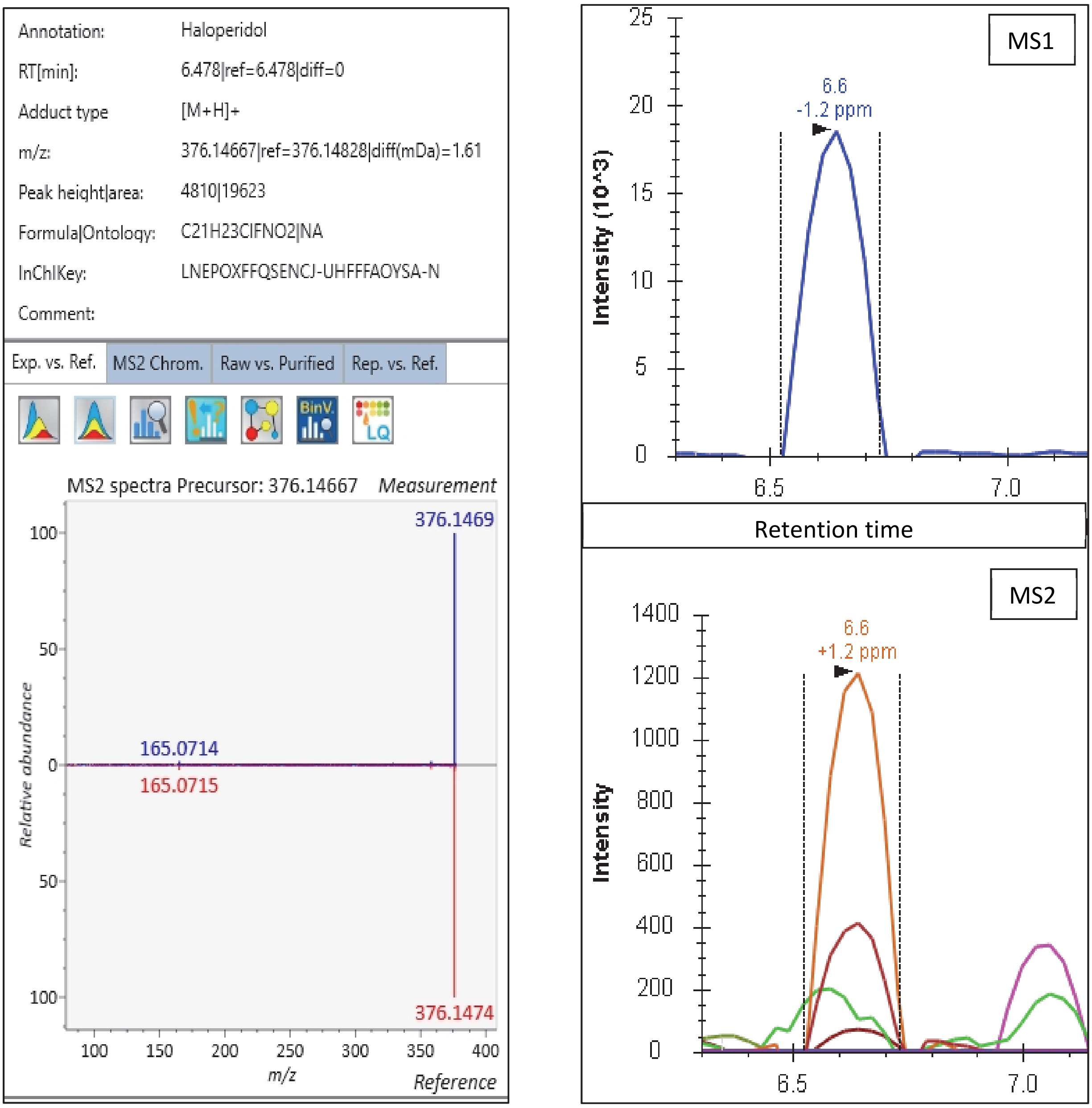

Figure S1. Continue

(H) DDA DIA

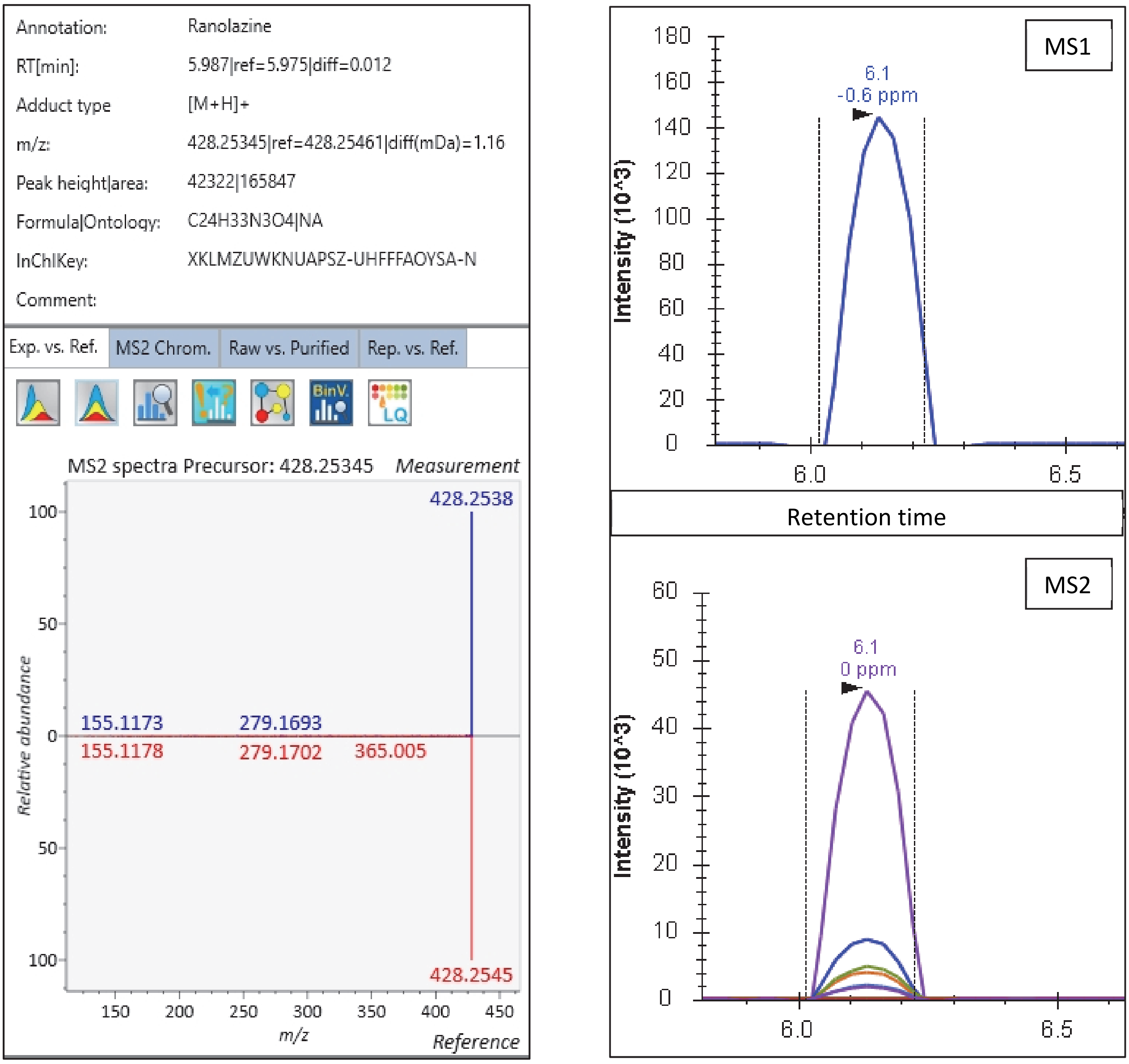

Figure S1. Continue

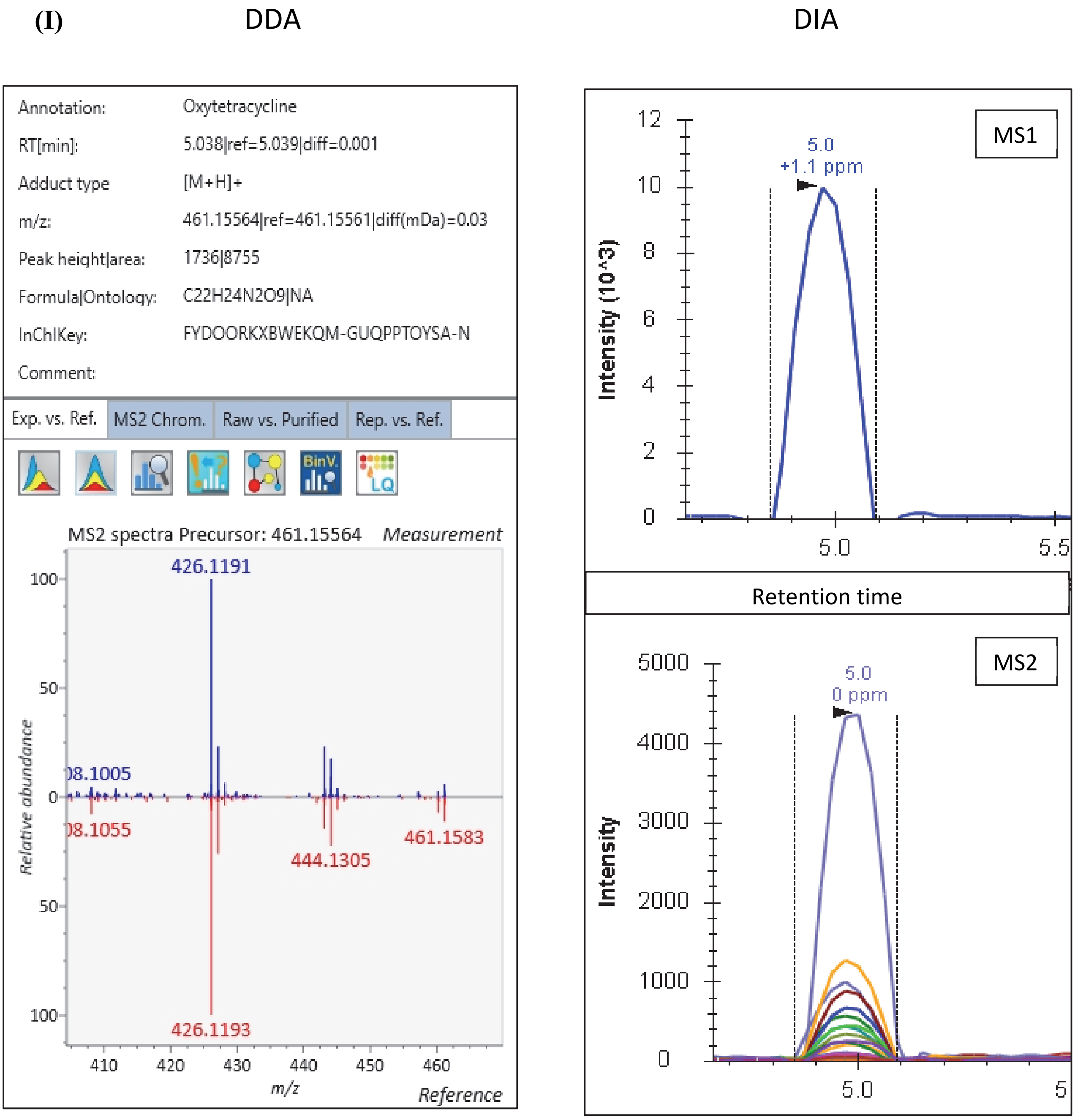

Figure S1. Continue

(J) DDA DIA

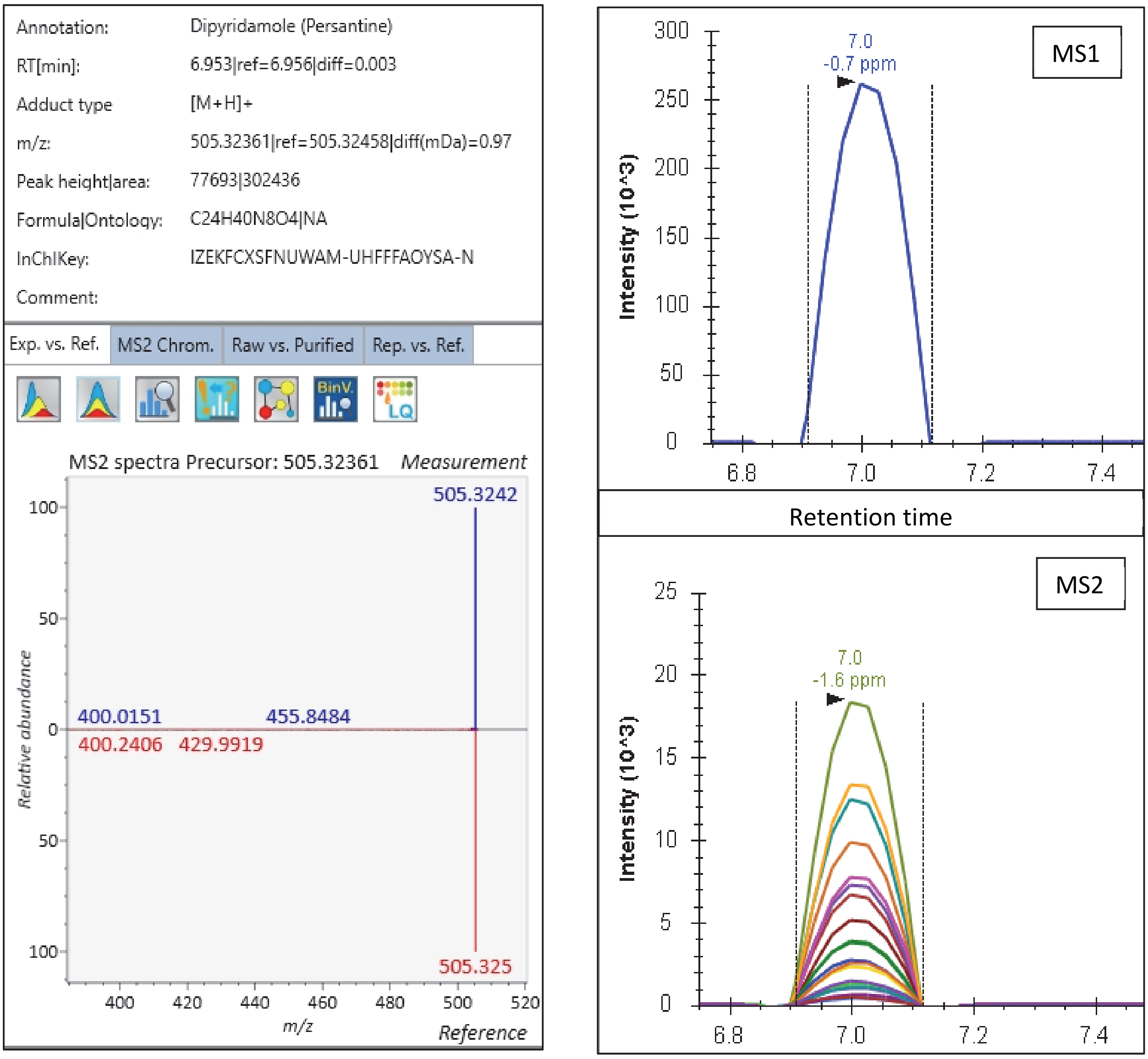

Figure S1. Identification of all drugs (**A**) Diphenhydramine; (**B**) Atropine; (**C**) Duloxetine; (**D**) Phenylbutazone; (**E**) Ranitidine; (**F**) Finasteride; (**G**) Haloperidol; (**H**) Ranolazine; (**I**) Oxytetracycline; (**J**) Dipyridamole have done by using MS-DIAL software in DDA mode. All identified and matched MS2 spectral information under 5ppm mass difference has given below. A transition list generated from all drugs spectral information and extracted in DIA mode using Skyline software.

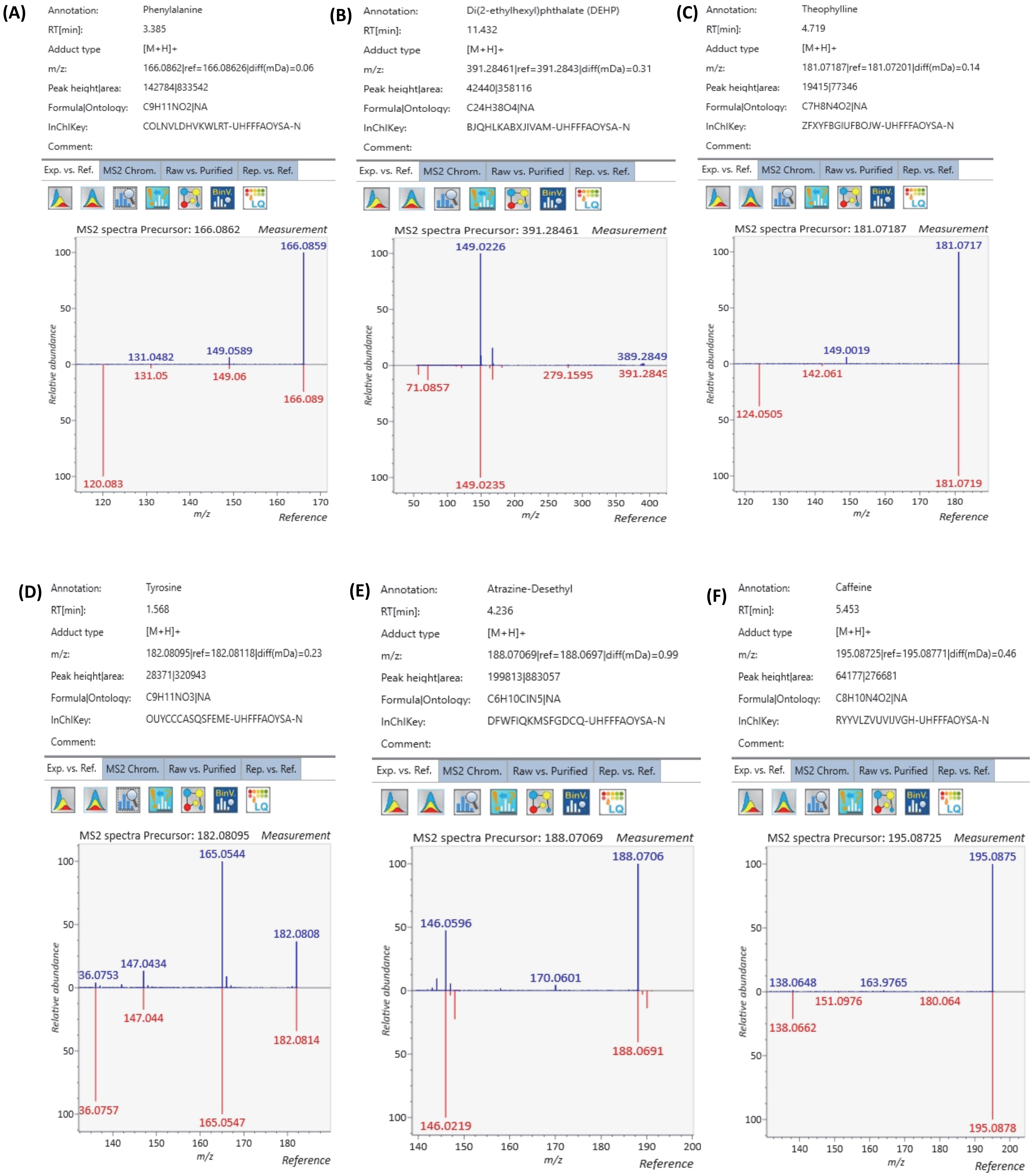
(A)
Annotation: Phenylalanine
RT[min]: 3.385
Adduct type [M+H]+
m/z: 166.0862|ref=166.08626|diff(mDa)=0.06
Peak height|area: 142784|833542
Formula|Ontology: C9H11NO2|NA
InChIKey: COLNVLDHVKWLRT-UHFFFAOYSA-N
Comment:
Exp. vs. Ref.
MS2 Chrom.
Raw vs. Purified
Rep. vs. Ref.
MS2 spectra Precursor: 166.0862
Measurement
166.0859
131.0482
149.0589
131.05
149.06
166.089
120.083
Relative abundance
m/z
Reference
(B)
Annotation: Di(2-ethylhexyl)phthalate (DEHP)
RT[min]: 11.432
Adduct type [M+H]+
m/z: 391.28461|ref=391.2843|diff(mDa)=0.31
Peak height|area: 42440|358116
Formula|Ontology: C24H38O4|NA
InChIKey: BJQHLKABXJIVAM-UHFFFAOYSA-N
Comment:
MS2 spectra Precursor: 391.28461
149.0226
389.2849
71.0857
279.1595
391.2849
149.0235
(C)
Annotation: Theophylline
RT[min]: 4.719
Adduct type [M+H]+
m/z: 181.07187|ref=181.07201|diff(mDa)=0.14
Peak height|area: 19415|77346
Formula|Ontology: C7H8N4O2|NA
InChIKey: ZFXYFBGIUFBOJW-UHFFFAOYSA-N
Comment:
MS2 spectra Precursor: 181.07187
181.0717
149.0019
142.061
124.0505
181.0719
(D)
Annotation: Tyrosine
RT[min]: 1.568
Adduct type [M+H]+
m/z: 182.08095|ref=182.08118|diff(mDa)=0.23
Peak height|area: 28371|320943
Formula|Ontology: C9H11NO3|NA
InChIKey: OUYCCCASQSFEME-UHFFFAOYSA-N
Comment:
MS2 spectra Precursor: 182.08095
165.0544
182.0808
147.0434
36.0753
147.044
182.0814
36.0757
165.0547
(E)
Annotation: Atrazine-Desethyl
RT[min]: 4.236
Adduct type [M+H]+
m/z: 188.07069|ref=188.0697|diff(mDa)=0.99
Peak height|area: 199813|883057
Formula|Ontology: C6H10ClN5|NA
InChIKey: DFWFIQKMSFGDCQ-UHFFFAOYSA-N
Comment:
MS2 spectra Precursor: 188.07069
188.0706
146.0596
170.0601
188.0691
146.0219
(F)
Annotation: Caffeine
RT[min]: 5.453
Adduct type [M+H]+
m/z: 195.08725|ref=195.08771|diff(mDa)=0.46
Peak height|area: 64177|276681
Formula|Ontology: C8H10N4O2|NA
InChIKey: RYYVLZVUVIJVGH-UHFFFAOYSA-N
Comment:
MS2 spectra Precursor: 195.08725
195.0875
138.0648
163.9765
151.0976
180.064
138.0662
195.0878

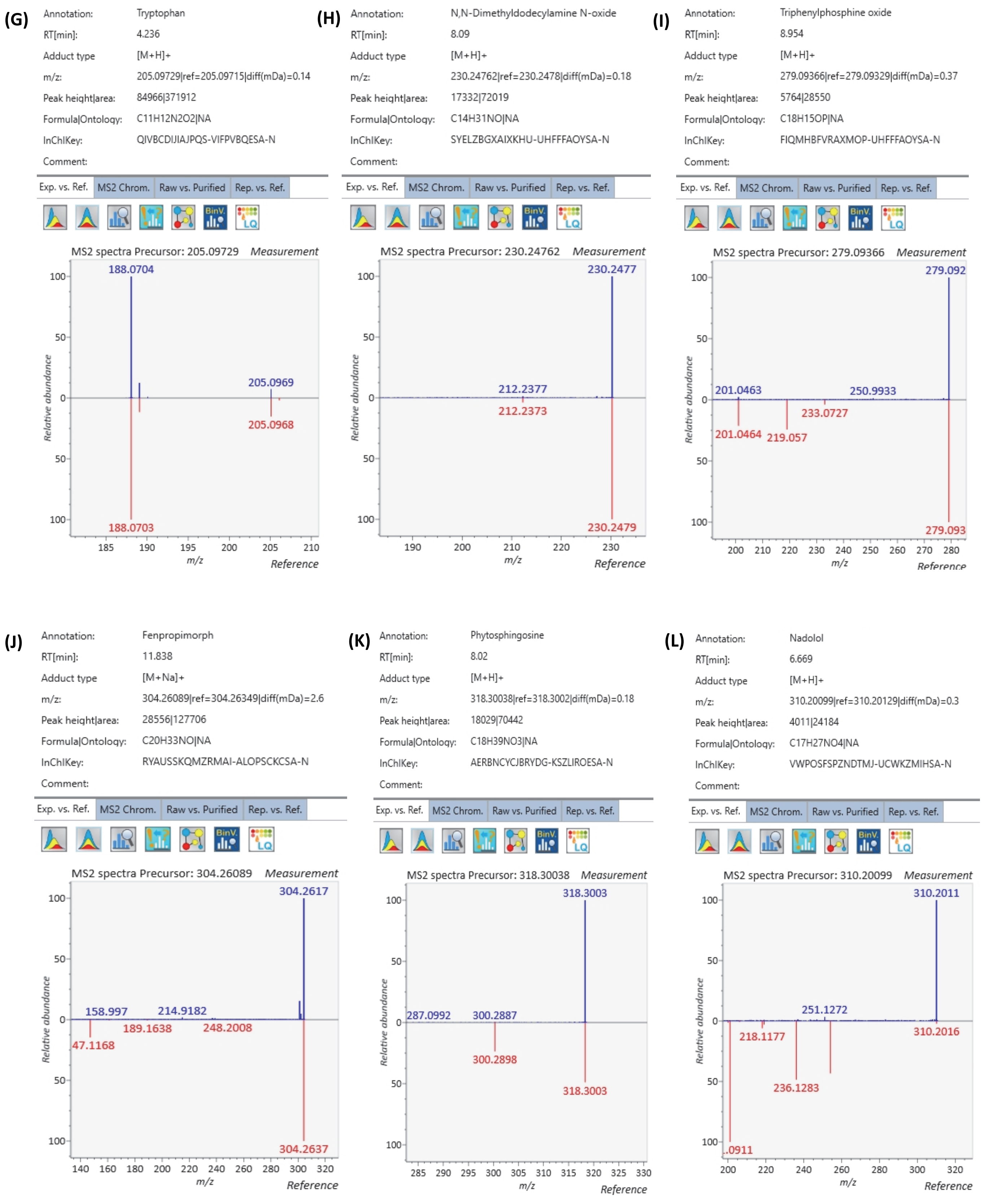
(G)
Annotation: Tryptophan
RT[min]: 4.236
Adduct type [M+H]+
m/z: 205.09729|ref=205.09715|diff(mDa)=0.14
Peak height|area: 84966|371912
Formula|Ontology: C11H12N2O2|NA
InChIKey: QIVBCDIJIAJPQS-VIFPVBQESA-N
Comment:
Exp. vs. Ref.
MS2 Chrom.
Raw vs. Purified
Rep. vs. Ref.
MS2 spectra Precursor: 205.09729 Measurement
188.0704
205.0969
205.0968
188.0703
Relative abundance
m/z
Reference
(H)
Annotation: N,N-Dimethyldodecylamine N-oxide
RT[min]: 8.09
Adduct type [M+H]+
m/z: 230.24762|ref=230.2478|diff(mDa)=0.18
Peak height|area: 17332|72019
Formula|Ontology: C14H31NO|NA
InChIKey: SYELZBGXAIXKHU-UHFFFAOYSA-N
Comment:
MS2 spectra Precursor: 230.24762 Measurement
230.2477
212.2377
212.2373
230.2479
(I)
Annotation: Triphenylphosphine oxide
RT[min]: 8.954
Adduct type [M+H]+
m/z: 279.09366|ref=279.09329|diff(mDa)=0.37
Peak height|area: 5764|28550
Formula|Ontology: C18H15OP|NA
InChIKey: FIQMHBFVRAXMOP-UHFFFAOYSA-N
Comment:
MS2 spectra Precursor: 279.09366 Measurement
279.092
201.0463
250.9933
233.0727
201.0464
219.057
279.093
(J)
Annotation: Fenpropimorph
RT[min]: 11.838
Adduct type [M+Na]+
m/z: 304.26089|ref=304.26349|diff(mDa)=2.6
Peak height|area: 28556|127706
Formula|Ontology: C20H33NO|NA
InChIKey: RYAUSSKQMZRMAI-ALOPSCKCSA-N
Comment:
MS2 spectra Precursor: 304.26089 Measurement
304.2617
158.997
214.9182
189.1638
248.2008
47.1168
304.2637
(K)
Annotation: Phytosphingosine
RT[min]: 8.02
Adduct type [M+H]+
m/z: 318.30038|ref=318.3002|diff(mDa)=0.18
Peak height|area: 18029|70442
Formula|Ontology: C18H39NO3|NA
InChIKey: AERBNCYCJBRYDG-KSZLIROESA-N
Comment:
MS2 spectra Precursor: 318.30038 Measurement
318.3003
287.0992
300.2887
300.2898
318.3003
(L)
Annotation: Nadolol
RT[min]: 6.669
Adduct type [M+H]+
m/z: 310.20099|ref=310.20129|diff(mDa)=0.3
Peak height|area: 4011|24184
Formula|Ontology: C17H27NO4|NA
InChIKey: VWPOSFSPZNDTMJ-UCWKZMIHSA-N
Comment:
MS2 spectra Precursor: 310.20099 Measurement
310.2011
251.1272
218.1177
310.2016
236.1283
.0911

(M)

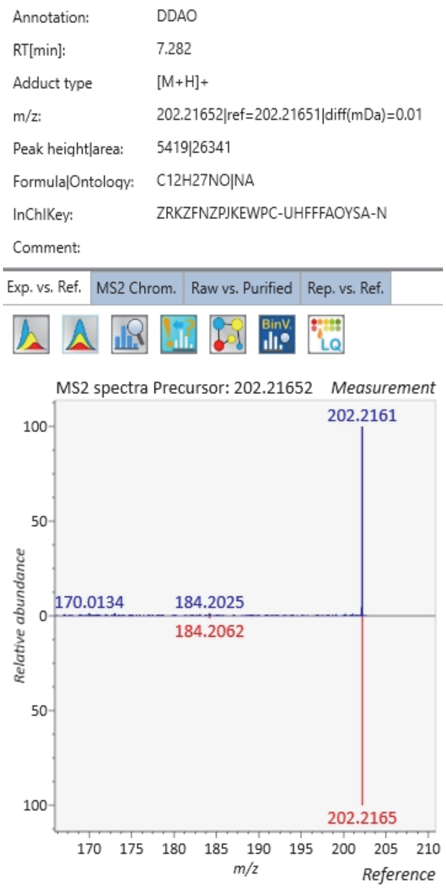
Annotation: DDAO
RT[min]: 7.282
Adduct type [M+H]+
m/z: 202.21652|ref=202.21651|diff(mDa)=0.01
Peak height|area: 5419|26341
Formula|Ontology: C12H27NO|NA
InChIKey: ZRKZFNZPJKEWPC-UHFFFAOYSA-N
Comment:
Exp. vs. Ref.
MS2 Chrom.
Raw vs. Purified
Rep. vs. Ref.
MS2 spectra Precursor: 202.21652
Measurement
202.2161
170.0134
184.2025
184.2062
202.2165
Relative abundance
m/z
Reference

(N)

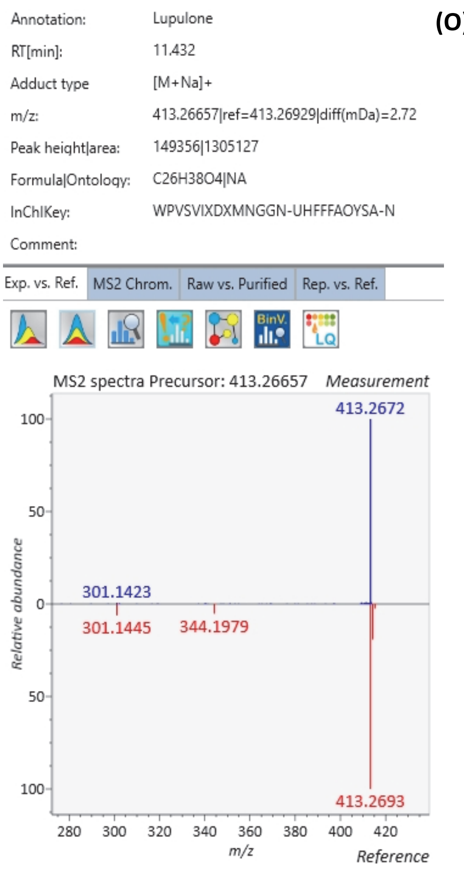
Annotation: Lupulone
RT[min]: 11.432
Adduct type [M+Na]+
m/z: 413.26657|ref=413.26929|diff(mDa)=2.72
Peak height|area: 149356|1305127
Formula|Ontology: C26H38O4|NA
InChIKey: WPVSVIXDXMNGGN-UHFFFAOYSA-N
Comment:
Exp. vs. Ref.
MS2 Chrom.
Raw vs. Purified
Rep. vs. Ref.
MS2 spectra Precursor: 413.26657
Measurement
413.2672
301.1423
301.1445
344.1979
413.2693
Relative abundance
m/z
Reference

(O)

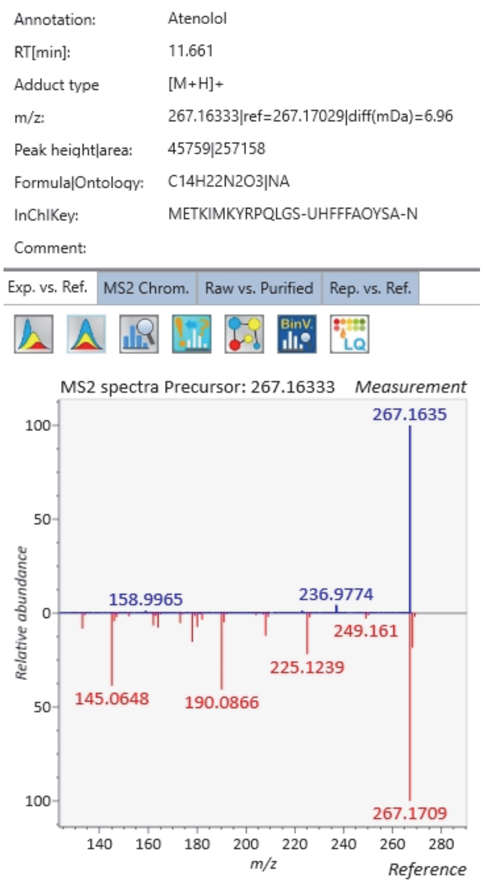
Annotation: Atenolol
RT[min]: 11.661
Adduct type [M+H]+
m/z: 267.16333|ref=267.17029|diff(mDa)=6.96
Peak height|area: 45759|257158
Formula|Ontology: C14H22N2O3|NA
InChIKey: METKIMKYRPQLGS-UHFFFAOYSA-N
Comment:
Exp. vs. Ref.
MS2 Chrom.
Raw vs. Purified
Rep. vs. Ref.
MS2 spectra Precursor: 267.16333
Measurement
267.1635
158.9965
236.9774
249.161
225.1239
145.0648
190.0866
267.1709
Relative abundance
m/z
Reference

(P)

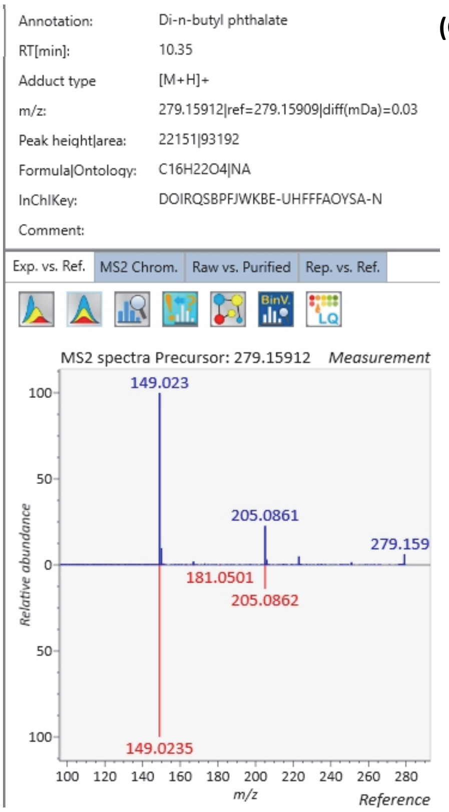
Annotation: Di-n-butyl phthalate
RT[min]: 10.35
Adduct type [M+H]+
m/z: 279.15912|ref=279.15909|diff(mDa)=0.03
Peak height|area: 22151|93192
Formula|Ontology: C16H22O4|NA
InChIKey: DOIRQSBPFJWKBE-UHFFFAOYSA-N
Comment:
Exp. vs. Ref.
MS2 Chrom.
Raw vs. Purified
Rep. vs. Ref.
MS2 spectra Precursor: 279.15912
Measurement
149.023
205.0861
279.159
181.0501
205.0862
149.0235
Relative abundance
m/z
Reference

(Q)

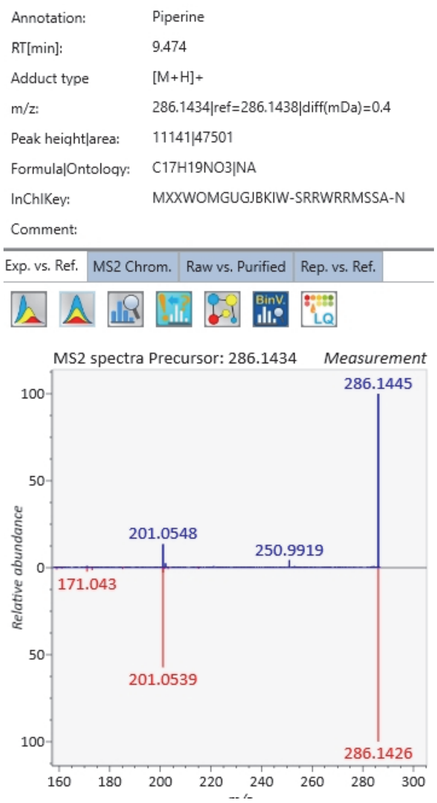
Annotation: Piperine
RT[min]: 9.474
Adduct type [M+H]+
m/z: 286.1434|ref=286.1438|diff(mDa)=0.4
Peak height|area: 11141|47501
Formula|Ontology: C17H19NO3|NA
InChIKey: MXXWOMGUGJBKIW-SRRWRRMSSA-N
Comment:
Exp. vs. Ref.
MS2 Chrom.
Raw vs. Purified
Rep. vs. Ref.
MS2 spectra Precursor: 286.1434
Measurement
286.1445
201.0548
250.9919
171.043
201.0539
286.1426
Relative abundance

(R)

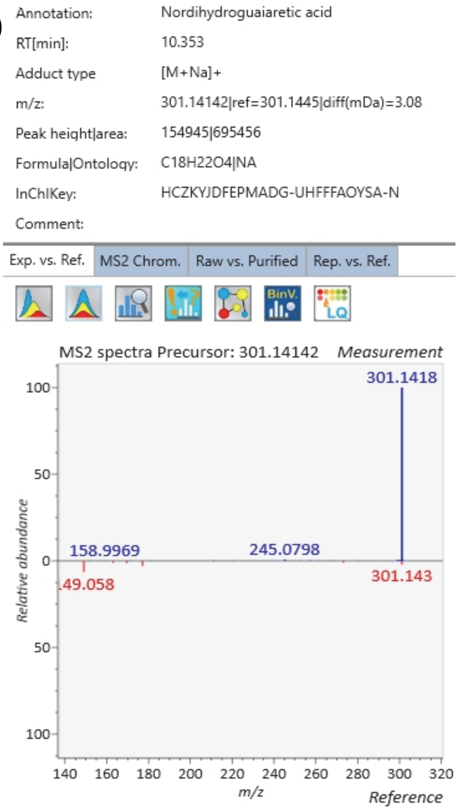
Annotation: Nordihydroguaiaretic acid
RT[min]: 10.353
Adduct type [M+Na]+
m/z: 301.14142|ref=301.1445|diff(mDa)=3.08
Peak height|area: 154945|695456
Formula|Ontology: C18H22O4|NA
InChIKey: HCZKYJDFEPMADG-UHFFFAOYSA-N
Comment:
Exp. vs. Ref.
MS2 Chrom.
Raw vs. Purified
Rep. vs. Ref.
MS2 spectra Precursor: 301.14142
Measurement
301.1418
158.9969
245.0798
149.058
301.143
Relative abundance
m/z
Reference

(S)

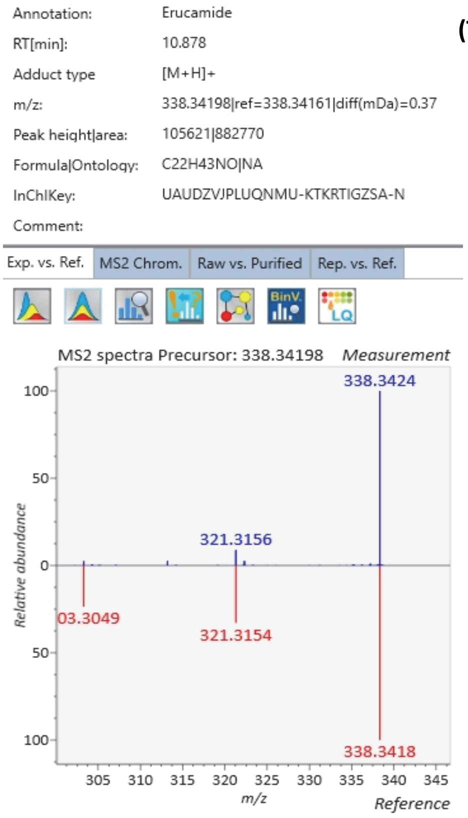
Annotation: Erucamide
RT[min]: 10.878
Adduct type [M+H]+
m/z: 338.34198|ref=338.34161|diff(mDa)=0.37
Peak height|area: 105621|882770
Formula|Ontology: C22H43NO|NA
InChIKey: UAUDZVJPLUQNMU-KTKRTIGZSA-N
Comment:
Exp. vs. Ref.
MS2 Chrom.
Raw vs. Purified
Rep. vs. Ref.
MS2 spectra Precursor: 338.34198
Measurement
338.3424
321.3156
03.3049
321.3154
338.3418
Relative abundance
m/z
Reference

(T)

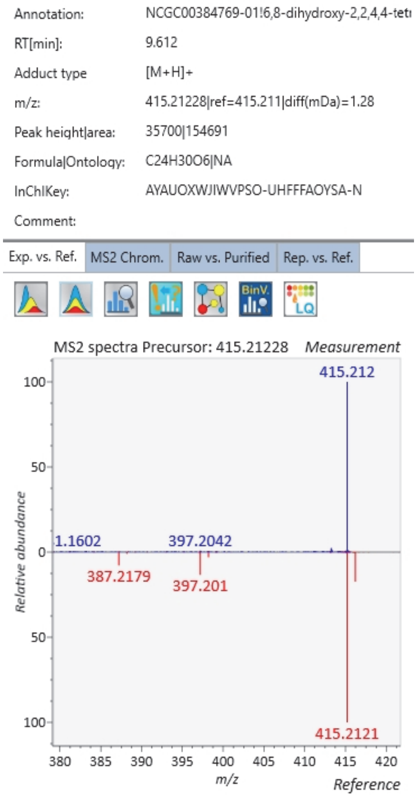
Annotation: NCGC00384769-01!6,8-dihydroxy-2,2,4,4-tet
RT[min]: 9.612
Adduct type [M+H]+
m/z: 415.21228|ref=415.211|diff(mDa)=1.28
Peak height|area: 35700|154691
Formula|Ontology: C24H30O6|NA
InChIKey: AYAUOXWJIWVPSO-UHFFFAOYSA-N
Comment:
Exp. vs. Ref.
MS2 Chrom.
Raw vs. Purified
Rep. vs. Ref.
MS2 spectra Precursor: 415.21228
Measurement
415.212
1.1602
397.2042
387.2179
397.201
415.2121
Relative abundance
m/z
Reference

(U)

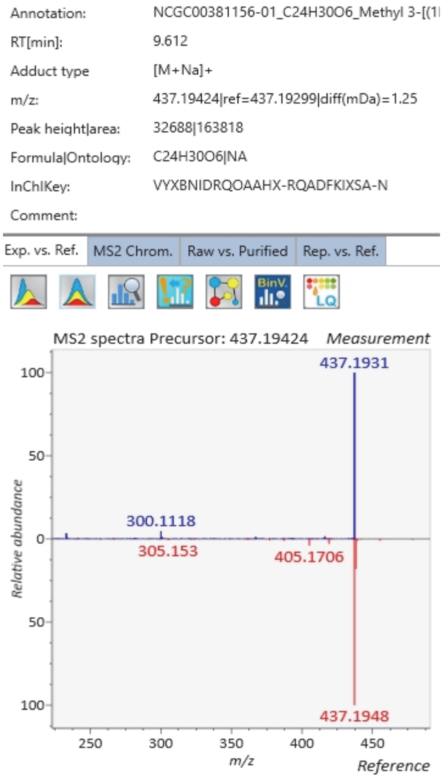
Annotation: NCGC00381156-01_C24H30O6_Methyl 3-[(1
RT[min]: 9.612
Adduct type [M+Na]+
m/z: 437.19424|ref=437.19299|diff(mDa)=1.25
Peak height|area: 32688|163818
Formula|Ontology: C24H30O6|NA
InChIKey: VYXBNIDRQOAAHX-RQADFKIXSA-N
Comment:
Exp. vs. Ref.
MS2 Chrom.
Raw vs. Purified
Rep. vs. Ref.
MS2 spectra Precursor: 437.19424
Measurement
437.1931
300.1118
305.153
405.1706
437.1948
Relative abundance
m/z
Reference

(V)

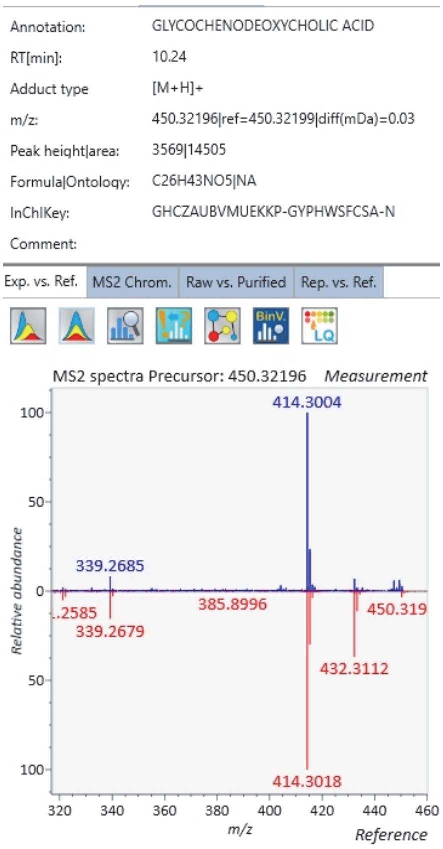
Annotation: GLYCOCHENODEOXYCHOLIC ACID
RT[min]: 10.24
Adduct type [M+H]+
m/z: 450.32196|ref=450.32199|diff(mDa)=0.03
Peak height|area: 3569|14505
Formula|Ontology: C26H43NO5|NA
InChIKey: GHCZAUBVMUEKKP-GYPHWSFCSA-N
Comment:
Exp. vs. Ref.
MS2 Chrom.
Raw vs. Purified
Rep. vs. Ref.
MS2 spectra Precursor: 450.32196
Measurement
414.3004
339.2685
.2585
339.2679
385.8996
450.319
432.3112
414.3018
Relative abundance
m/z
Reference

(W)

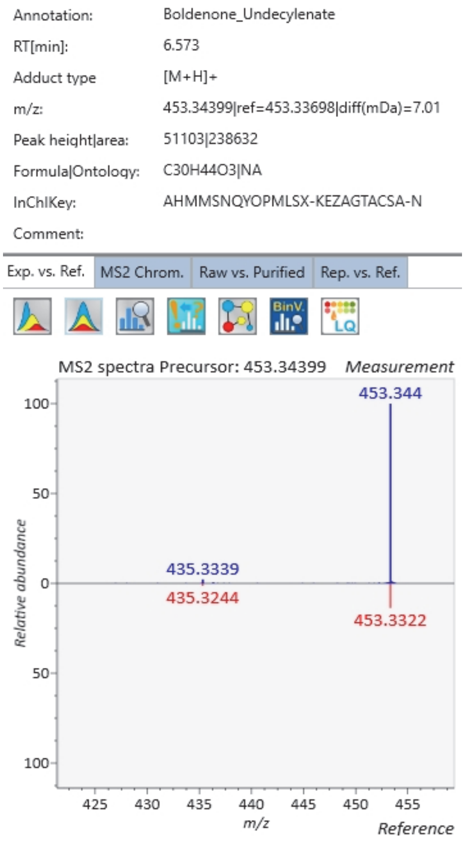
Annotation: Boldenone_Undecylenate
RT[min]: 6.573
Adduct type [M+H]+
m/z: 453.34399|ref=453.33698|diff(mDa)=7.01
Peak height|area: 51103|238632
Formula|Ontology: C30H44O3|NA
InChIKey: AHMMSNQYOPMLSX-KEZAGTACSA-N
Comment:
Exp. vs. Ref.
MS2 Chrom.
Raw vs. Purified
Rep. vs. Ref.
MS2 spectra Precursor: 453.34399
Measurement
453.344
435.3339
435.3244
453.3322
Relative abundance
m/z
Reference

Figure S2. Identified serum metabolites by their MS2 spectra in DDA mode using MS-DIAL software. The software-generated subfigures contain exact mass, retention time and fragments mass of serum metabolites. (**A**) Phenylalanine; (**B**) Di(2-ethylhexyl) phthalate (DEHP); (**C**) Theophylline; (**D**) Tyrosine; (**E**) Atrazine-desethyl; (**F**) Caffeine; (**G**) Tryptophan; (**H**) N, N- Dimethyl dodecyl amine N-oxide; (**I**) Triphenylphosphine oxide; (**J**) Fenpropimorph; (**K**) Phytosphingosine; (**L**) Nadolol; (**M**) DDAO; (**N**) Lupulone; (**O**) Atenolol; (**P**) Di-n-butyl phthalate; (**Q**) Piperine; (**R**) Nordihydroguaiaretic Acid; (**S**) Erucamide; (**T**) NCGC00384769-01; (**U**) NCGC00381156-01; (**V**) Glycochenodeoxycholic Acid; (**W**) Boldenone-Undecylenate.

(**A**) DIPHENHYDRAMINE

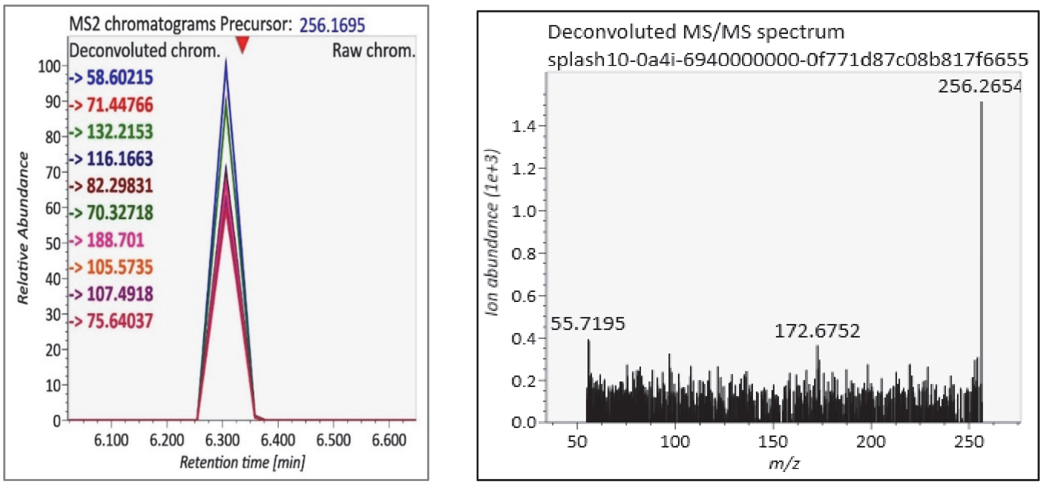

(**B**) PHENYLBUTAZONE

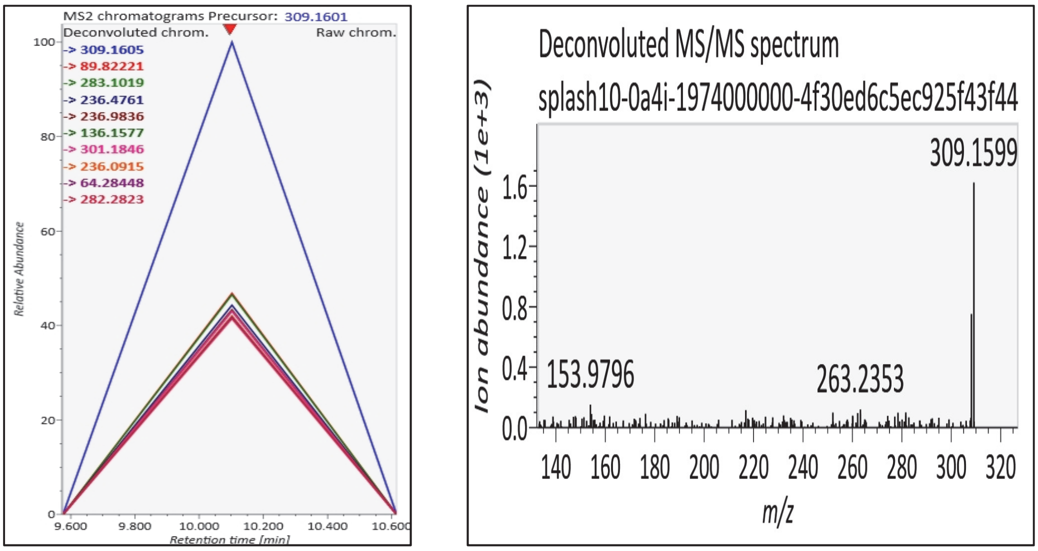

(**C**) RANITIDINE

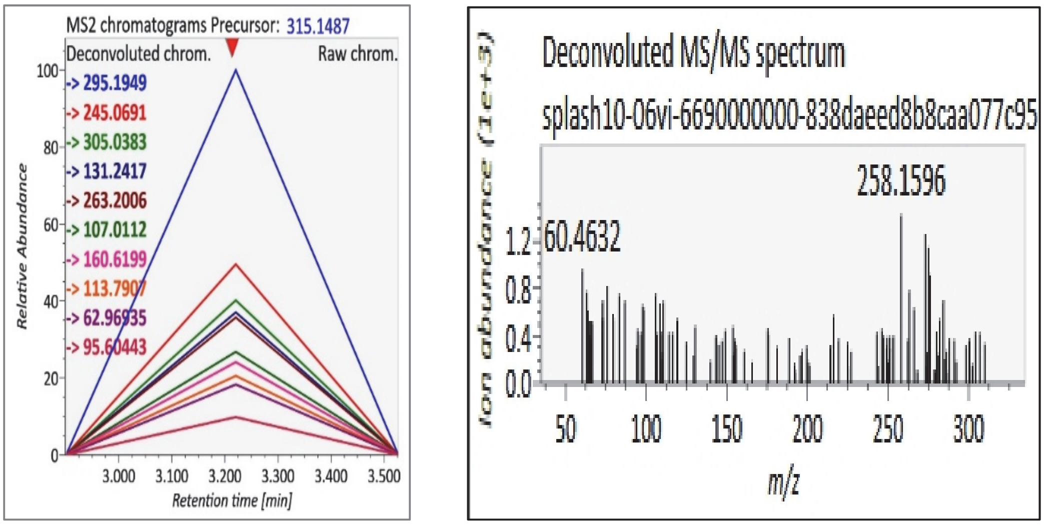

(**D**) RANOLAZINE

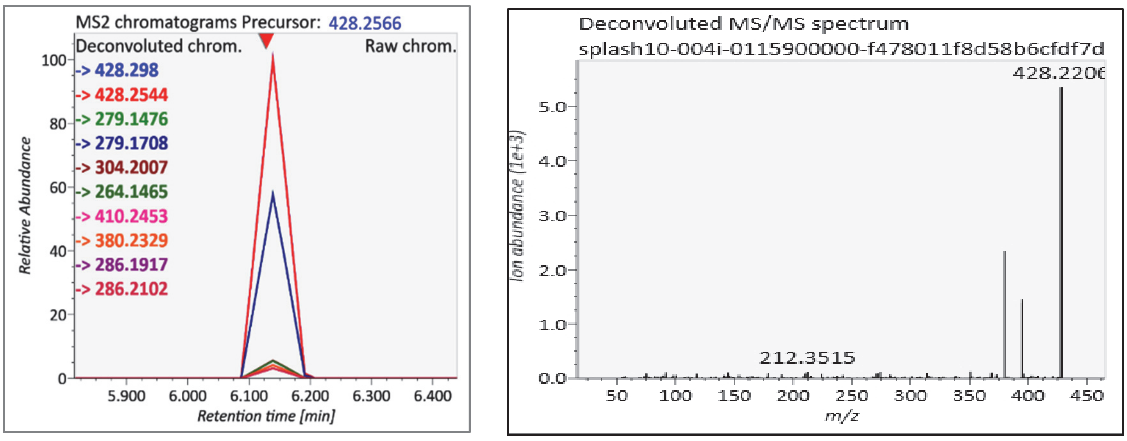

(**E**) FINASTERIDE

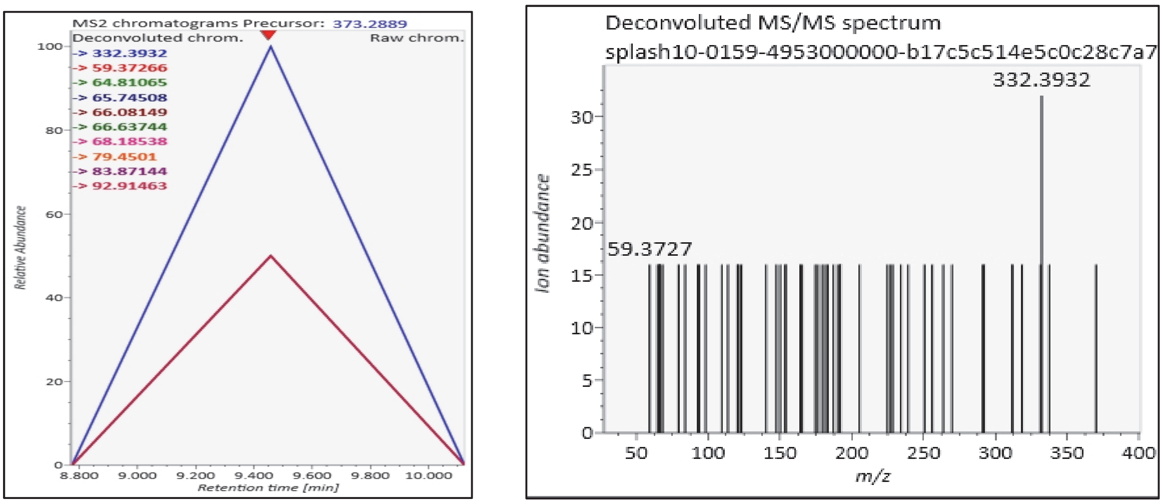

Figure S3. DIA MS^2 deconvolution results of drug standards were not satisfying for in MS-DIAL. (**A**) Diphenhydramine; (**B**) Phenylbutazone; (**C**) Ranitidine; (**D**) Ranolazine; (**E**) Finasteride.

(A) PHENYLALANINE **(B) ATRAZINE-DESETHYL** **(C)TRYPTOPHAN** **(D) THEOPHYLLINE**

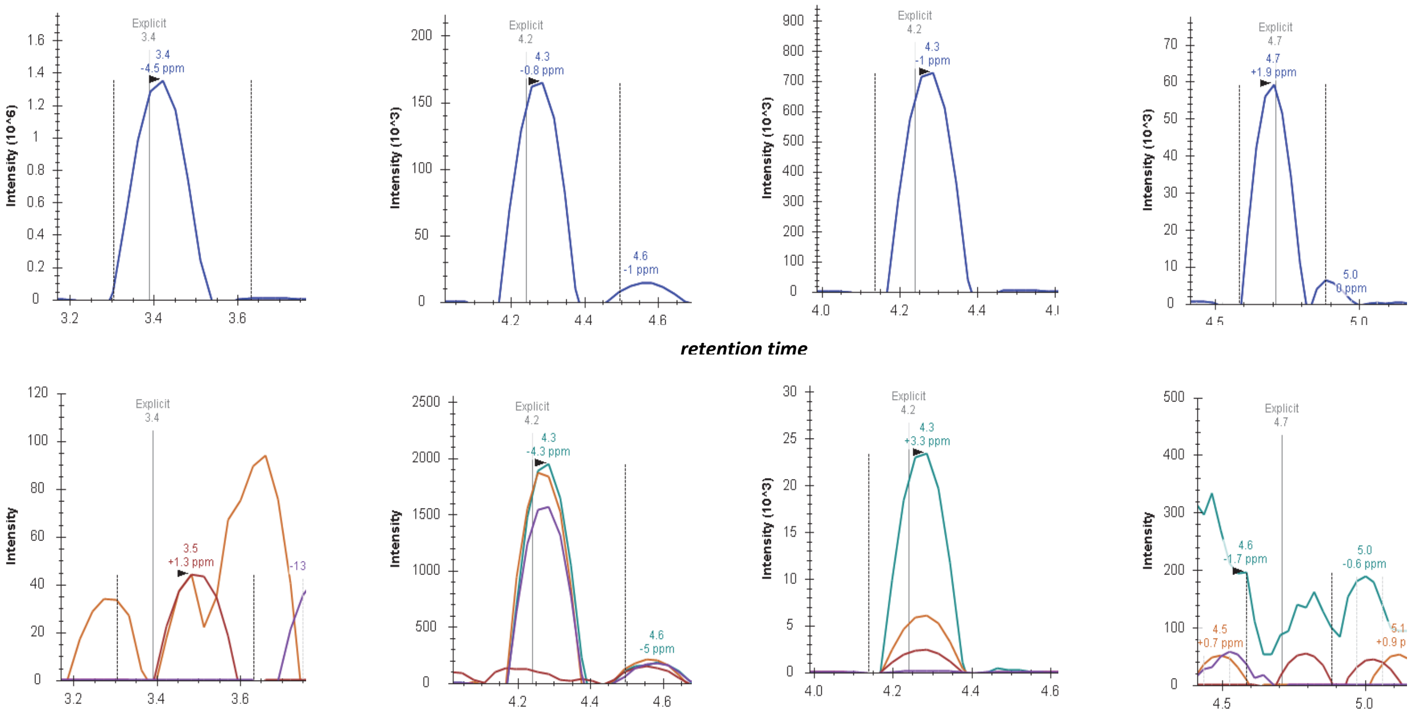

(E) CAFFEINE **(F) BOLDENONE_UNDECYLENATE** **(G) NADOLOL** **(H) DDAO**

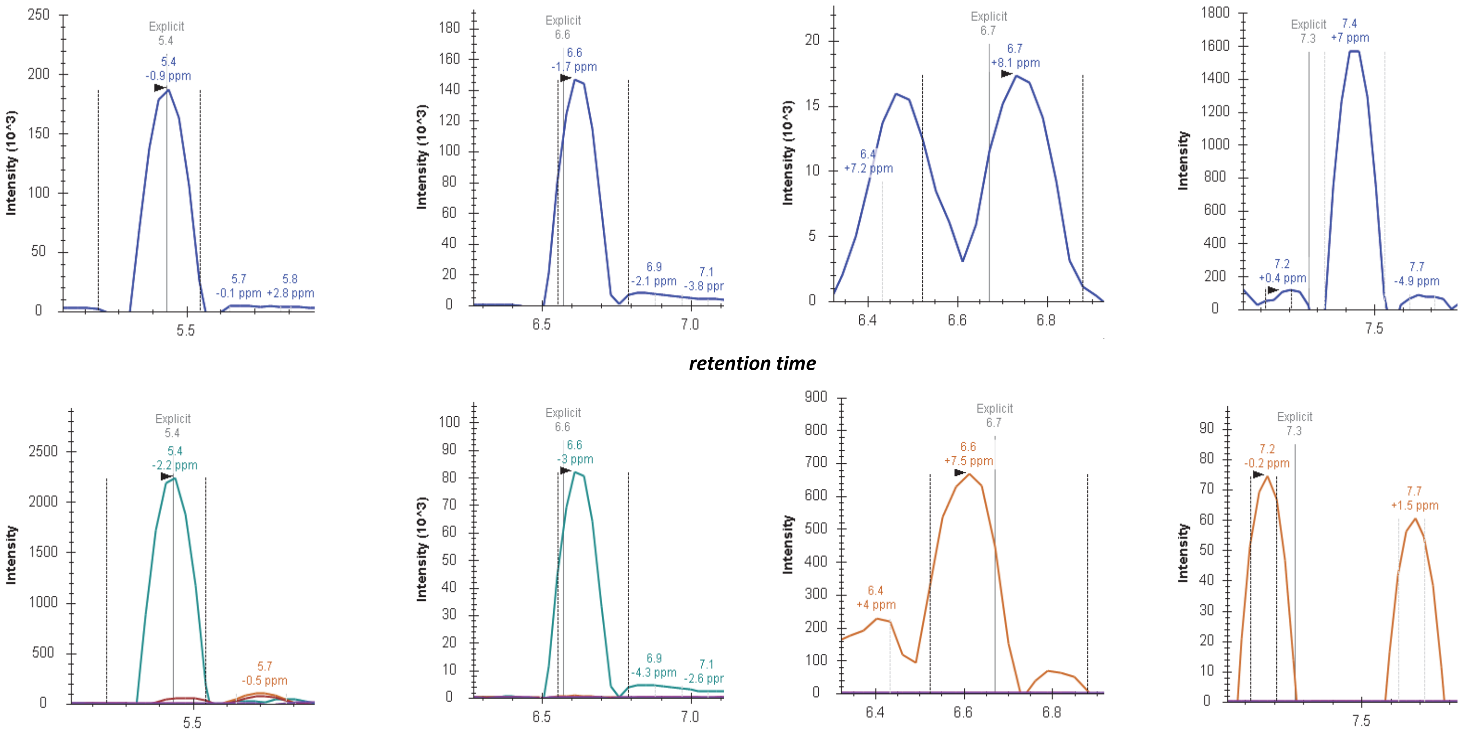

(I) PHYTOSPHINGOSINE (J) N, N-DIMETHYLDODECYLAMINE N-OXIDE (K) PIPERINE (L) NCGC00384769-01

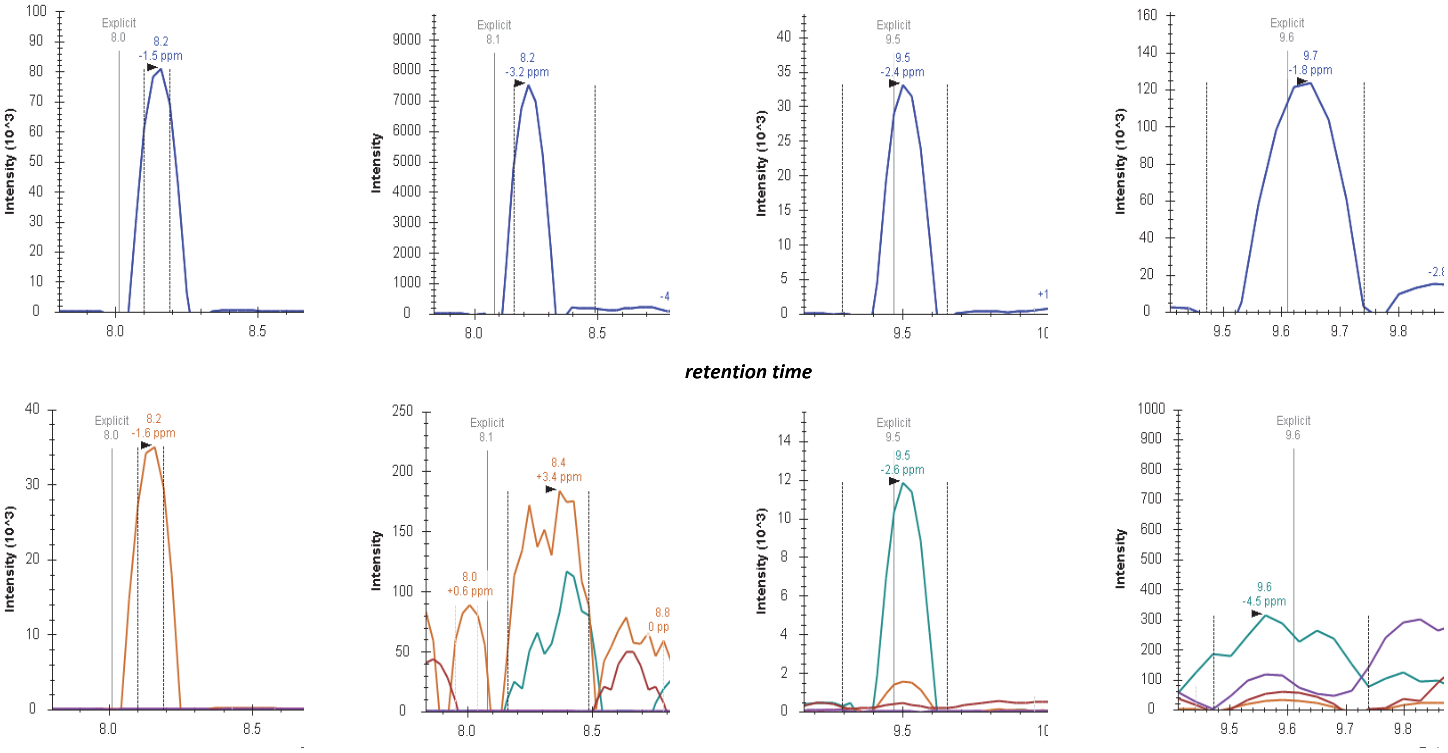

(M) NCGC00381156-01 (N) GLYCOCHENODEOXYCHOLIC ACID (O) DI-N-BUTYL PHTHALATE (P) NORDIHYDROGUAIARETIC ACID

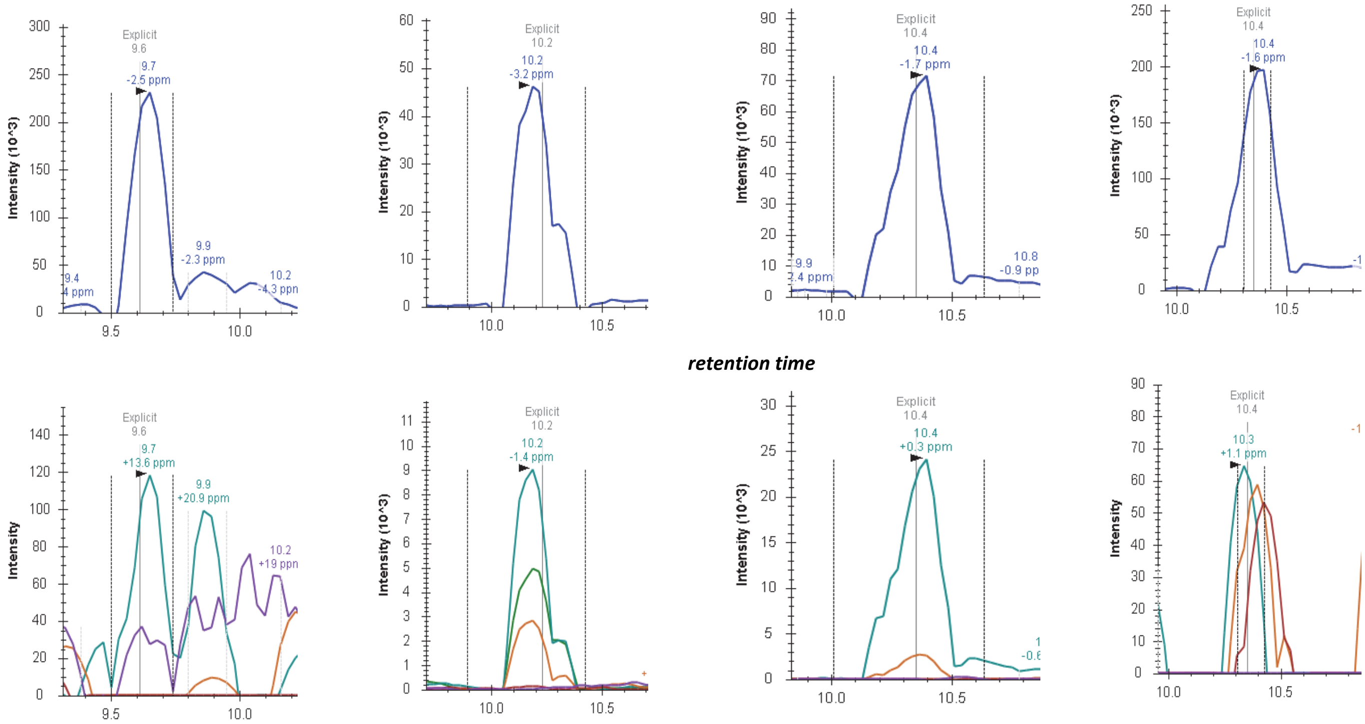

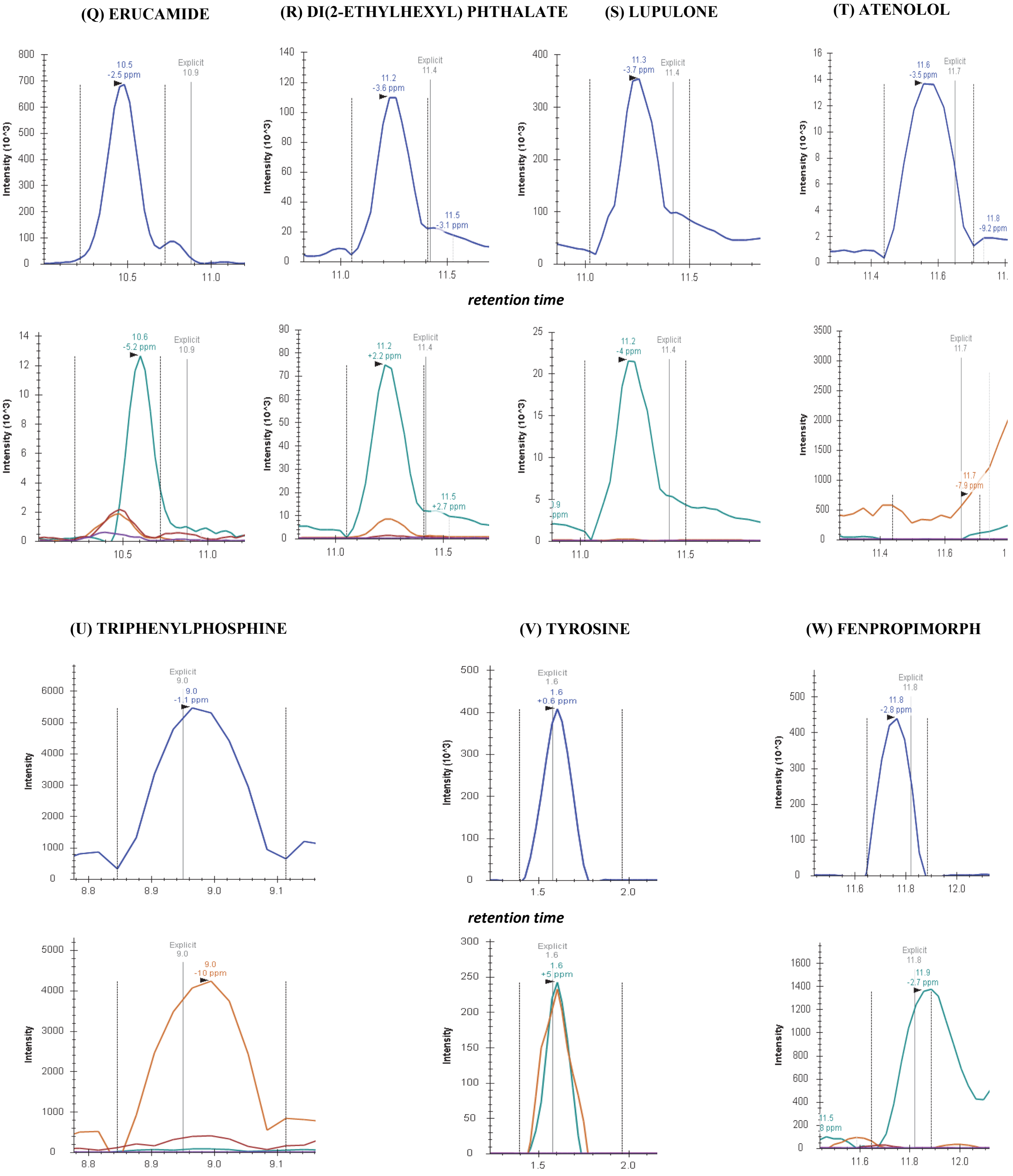

Figure S4. Identified serum metabolites deconvoluted MS1 (top) and MS2 (bottom) spectra by DIA mode in Skyline. (**A**) Phenylalanine; (**B**) Atrazine-Desethyl; (**C**) Tryptophan; (**D**) Theophylline; (**E**) Caffeine; (**F**) Boldenone-Undecylenate; (**G**) Nadolol; (**H**) DDAO; (**I**) Phytosphingosine; (**J**) N, N-Dimethyl-dodecyl-amine-N-Oxide; (**K**) Piperine; (**L**) NCGC00384769-01; (**M**) NCGC00381156-01; (**N**) Glycochenodeoxycholic Acid; (**O**) Di-N-Butyl Phthalate; (**P**) Nordihydroguaiaretic Acid; (**Q**)

Erucamide; (**R**) Di(2-Ethylhexyl) Phthalate; (**S**) Lupulone; (**T**) Atenolol; (**U**) Triphenylphosphine; (**V**) Tyrosine; (**W**) Fenpropimorph.
